# Novel Insights into *E. coli*’s Hexuronate Metabolism: KduI Facilitates the Conversion of Galacturonate and Glucuronate under Osmotic Stress Conditions

**DOI:** 10.1371/journal.pone.0056906

**Published:** 2013-02-21

**Authors:** Monique Rothe, Carl Alpert, Gunnar Loh, Michael Blaut

**Affiliations:** Department of Gastrointestinal Microbiology, German Institute of Human Nutrition Potsdam-Rehbrücke, Nuthetal, Germany; University of Groningen, Groningen Institute for Biomolecular Sciences and Biotechnology, The Netherlands

## Abstract

Using a gnotobiotic mouse model, we previously observed the upregulation of 2-deoxy-D-gluconate 3-dehydrogenase (KduD) in intestinal *E. coli* of mice fed a lactose-rich diet and the downregulation of this enzyme and of 5-keto 4-deoxyuronate isomerase (KduI) on a casein-rich diet. The present study aimed to define the role of the so far poorly characterized *E. coli* proteins KduD and KduI *in vitro*. Galacturonate and glucuronate induced *kduD* and *kduI* gene expression 3-fold and 7 to 11-fold, respectively, under aerobic conditions as well as 9 to 20-fold and 19 to 54-fold, respectively, under anaerobic conditions. KduI facilitated the breakdown of these hexuronates. In *E. coli*, galacturonate and glucuronate are normally degraded by UxaABC and UxuAB. However, osmotic stress represses the expression of the corresponding genes in an OxyR-dependent manner. When grown in the presence of galacturonate or glucuronate, *kduID*-deficient *E. coli* had a 30% to 80% lower maximal cell density and 1.5 to 2-fold longer doubling times under osmotic stress conditions than wild type *E. coli*. Growth on lactose promoted the intracellular formation of hexuronates, which possibly explain the induction of KduD on a lactose-rich diet. These results indicate a novel function of KduI and KduD in *E. coli* and demonstrate the crucial influence of osmotic stress on the gene expression of hexuronate degrading enzymes.

## Introduction

The intestinal tract harbors a complex microbial community, whose composition is modulated by nutrition [1], [2], [3]. However, diet is also a crucial factor that influences the activity of the intestinal microbiota [1], [4], [5]. Microbiome analysis revealed an increase in genes encoding bacterial enzymes involved in amino acid degradation, carbohydrate catabolism, vitamin biosynthesis, and bile salt metabolism in response to a protein-rich host diet [6]. Using a gnotobiotic mouse model, we previously demonstrated that intestinal *Escherichia coli* K-12 (MG1655) modulates its metabolism in response to various diets [7].

The key finding in this study was the upregulation of OxyR-dependent proteins in intestinal *E. coli* from mice fed a lactose-rich diet and their essential role in bacterial adaptation to lactose-mediated osmotic stress [7]. In addition, we observed the diet-dependent expression of two *E. coli* proteins that have been identified [8], [9], [10] but whose roles in the *E. coli* metabolism are still obscure: namely, the 2-deoxy-D-gluconate 3-dehydrogenase (KduD), which was 2.4-fold upregulated in *E. coli* of mice on the lactose diet and 4.0-fold downregulated in *E. coli* of mice on the casein diet (both versus control [starch] diet), and the 5-keto 4-deoxyuronate isomerase (KduI), which was 8.3-fold lower in *E. coli* of mice fed the casein diet [7]. In the plant pathogen *Erwinia chrysanthemi* these enzymes are involved in pectin degradation [11], [12]. However, since *E. coli* K-12 lacks enzymes required for transport and catabolism of pectin or poly- and oligogalacturonates [13], it is not capable of degrading these carbohydrates. This study aimed to further characterize the role of KduI and KduD in *E. coli*. The results obtained indicate that KduI and KduD play a crucial role in the conversion of hexuronates in *E. coli* under osmotic stress conditions.

## Materials and Methods

### Ethics Statement

The protocol for the animal study was approved by the animal welfare committee of the “Landesamt für Verbraucherschutz, Landwirtschaft und Flurerneuerung, Referat Tierarzneimittelüberwachung, Tierschutz und Grenzveterinärdienst“, state of Brandenburg, Germany (approval number: 23-2347-8-25-2008). The experiments were performed according to the German guidelines for the care and use of animals in laboratory research (§8, Abs. 1, 25.05.1998).

### Animal Experiment

Three groups of 18 to 21 germfree C3H mice (Charles River), 9 to 12 weeks of age, were housed in a sterile Trexler type isolator (4 to 5 mice per cage) at constant room temperature of 22°C ±10%, air humidity of 55% ±10%, and a light/dark cycle of 12****h. The mice had free access to autoclaved water and either one of three sterilized semi-synthetic diets: a diet rich in starch, a lactose-rich diet, or a casein-rich diet (Table S1 in the supplementary material). The germfree status of the animals was confirmed prior to the initiation of the experiment by Gram staining and aerobic and anaerobic cultivation of fecal material [14]. Each mouse was inoculated with 1×10^7^ *E. coli* K-12 MG1655 cells by gastric gavage and killed 21 days after inoculation by cervical dislocation. The small intestinal, caecal, and colonic contents were collected and prepared for two-dimensional difference gel electrophoresis as described previously [7]. Contaminations were excluded by amplification and sequencing of the bacterial 16S rRNA genes of representative caecal samples with the primers 27-f (5′-AGA GTT TGA TCC TGG CTC AG-3′) and 1492-r (5′-TAC CTT GTT ACG ACT T-3′) [7], [15]. In this animal experiment we compared the proteomes of *E. coli* obtained from mice fed the lactose diet or the casein diet with those of mice fed the starch diet and identified differentially expressed proteins by electrospray-tandem mass spectrometry. One result of this study was the induction of some OxyR-dependent proteins by the lactose-rich diet [7]. In the present study two other differentially expressed proteins, namely KduI and KduD, were investigated for their possible role in the adaptation of intestinal *E. coli* to the lactose diet.

### Luciferase Reporter Gene Assays

To identify substances inducing *kduI* and *kduD* gene expression in intestinal *E. coli*, luciferase reporter gene assays were applied. Reporter gene constructs (p*kduIp::luxAB*, p*kduDp::luxAB*) were generated and validated as described previously [7]. The primers used are listed in Table 1. To measure promoter activity in response to the mouse diets, *E. coli* clones MG1655 or Δ*oxyR* carrying p*kduIp::luxAB* or p*kduDp::luxAB* were incubated in M9 minimal medium (Na_2_HPO_4_ [6 g/liter], KH_2_PO_4_ [3 g/liter], NaCl [0.5 g/liter], NH_4_Cl [1 g/liter], uracil [12.5 mg/liter], 1 mM MgSO_4_, 0.1 mM CaCl_2_) plus carbenicillin (50 µg/ml) in the presence of the pulverized mouse diets (1%) or the dietary components glucose, fructose, galactose (50 mM each), lactose (25 mM), or casaminoacids (1%). Cultures (10 ml medium, inoculated with one *E. coli* colony) were grown aerobically in 100-ml Erlenmeyer flasks or anaerobically in gassed Hungate tubes (80% nitrogen, 20% carbon dioxide) and shaken at 220 and 120 rpm, respectively, at 37°C for 16 h.

**Table 1:** Primers used for generation of luciferase reporter gene constructs.

Code	Amplified region	Primer	Reference or source
primer-*kduIp*	promoter region of *ahpCF*	CGGAATTCGCCAGGGTGTGGCATTGAC^a^	This work
		GCTCTAGAGTGCGCACTGTGGATGCTCT^b^	This work
primer-*kduDp*	promoter region of *kduD*	CGGAATTCTAACCTCACCAGTAACCGTCG^a^	This work
		GCTCTAGAACGACCGCAACTTTACCTTC^b^	This work
primer-*uxaCAp*	promoter region of *uxaCA*	CGTCTAGACAATTTCCAGAGTCCGA^a^	This work
		CGGAATTCGAAGATGTTAGTTACG^b^	This work
primer-*uxaBp*	promoter region of *uxaB*	CGTCTAGAATGGGTTCCCTTCTGA^a^	This work
		CGGAATTCATACGTGTCTGTATC^b^	This work
primer-*uxuABp*	promoter region of *uxuAB*	CGGAATTCGGTCAACCATTGTTGCGATG^a^	This work
		CGTCTAGAGACCGCAGCCGTGGCG^b^	This work
pKESTMR control	Integration site of pKESTMR	AAAGTGCCACCTGACGT^a^	Rothe et al. [7]
		GGGTTGGTATGTAAGCAA^b^	Rothe et al. [7]
oxyR control	Up−/down-stream of *oxyR*	GCTGCAATCGTGCCTCGACA^a^	Rothe et al. [7]
		TCGTCGGCATGAACGTGGG^b^	Rothe et al. [7]

a forward primer,

b reverse primer.

Cells were harvested by centrifugation at 10,000×g and 4°C for 10 min and bacterial pellets were resuspended in PBS (Na_2_HPO_4_ [80 g/liter], KCl [2 g/liter], Na_2_HPO_4_ [14.4 g/liter], KH_2_PO_4_ [2.4 g/liter], pH 7.4) containing chloramphenicol (30 µg/ml; Roth, Karlsruhe, Germany) to inhibit protein biosynthesis. Optical density at 600 nm (OD_600_) was determined (SmartSpec Plus spectrophotometer; Bio-Rad, Munich, Germany) and cell concentrations were adjusted to approximately 5×10^9^ cells/ml. Luminescence of 2.5×10^8^ cells in 50 µl was measured as described previously [7]. Relative luminescence values were calculated as follows: luminescence values for p*kduIp::luxAB* and p*kduDp::luxAB* were related to values measured in the presence of 50 mM glucose. Since glucose undergoes conversion by glycolytic enzymes, induction of *kduI* and *kduD* by glucose was not expected to happen.

To address the expression of the standard hexuronate degrading enzymes UxaABC and UxuAB, reporter genes (p*uxaCAp::luxAB*, p*uxaBp::luxAB*, p*uxuABp::luxAB*) were constructed as described above. To measure the expression of the corresponding genes in response to osmotic stress, *E. coli* MG1655 or Δ*oxyR* clones carrying p*uxaCAp::luxAB*, p*uxaBp::luxAB*, or p*uxuABp::luxAB* were precultured aerobically or anaerobically in LB-Lennox medium (Roth, Karlsruhe, Germany) plus carbenicillin (50 µg/ml) and inoculated at 5% into 75 ml (aerobic) or 200 ml (anaerobic) fresh LB-Lennox medium plus carbenicillin (50 µg/ml). Cells were grown for approximately 1.5 h to mid-exponential growth phase under shaking at 220 or 120 rpm. Cell suspensions grown on LB medium were centrifuged at 5,000×g and 4°C for 5 min, pelleted cells were washed twice with 20 ml PBS, and resuspended in M9 minimal medium. Cell concentrations were adjusted to 5×10^9^ cells/ml as described above.

To investigate the promoter activity in response to hexuronates with or without osmotic stress, 450 µl cell suspension was transferred to Eppendorf tubes and centrifuged at 5,000×g and 4°C for 5 min. The pellet was resuspended in M9 minimal medium (negative control), or M9 minimal medium containing galacturonate or glucuronate (50 mM each) with or without sucrose (25 to 400 mM), H_2_O_2_ (300 µM), or NaCl (400 mM) plus carbenicillin (50 µg/ml). Cells were incubated at 37°C for 90 min under shaking at 120 rpm either in sterile 12-well plates for analysis under aerobic conditions or in sterile, gassed Hungate tubes (80% nitrogen, 20% carbon dioxide) for analysis under anaerobic conditions. Preparation of cells for subsequent measurement of luminescence was done as described above. Relative luminescence values were calculated as follows: luminescence values for p*uxaCAp::luxAB*, p*uxaBp::luxAB*, and p*uxuABp::luxAB* were related to values measured in the presence of 50 mM galacturonate and glucuronate, respectively.

### Generation and Characterization of an *E. coli* Mutant Lacking *kduID*

The deletion mutant lacking *kduID* was constructed by replacing the chromosomal *kduID* gene sequence by a kanamycin resistance cassette (Figure S2 in the supplemental material) according to the technique of Datsenko and Wanner [16] as described previously [7]. Primers are listed in Table 2. To characterize the growth of the *kduID* deletion mutant, *E. coli* MG1655 and Δ*kduID* were precultured aerobically or anaerobically in 10 ml M9 minimal medium with galacturonate or glucuronate (50 mM each) and inoculated at 2.5×10^7^ cells/ml into fresh M9 minimal medium containing galacturonate or glucuronate (50 mM each) with or without sucrose (200 mM, 400 mM, or 700 mM). Mutant *E. coli* were precultured in the presence of kanamycin (50 µg/ml). Aerobic incubation at 37°C was done in transparent 12-well culture plates (1 ml per well; Sigma-Aldrich, Steinheim, Germany) in a Tecan Infinite F200 Pro microplate reader (Tecan Group Ltd., Männedorf, Switzerland) under constant shaking at 218 rpm for 45 h. For incubation under anaerobic conditions, inoculation of fresh media was done in sterile, gassed Hungate tubes (80% nitrogen, 20% carbon dioxide) and 1 ml culture aliquots were transferred to 12-well plates in an anaerobic chamber (80% nitrogen, 10% carbon dioxide, 10% hydrogen; Meintrup dws Laborgeräte, Lähden-Holte, Germany). To maintain anoxic conditions, 12-well plates were carefully covered with an adhesive film. Anaerobic conditions were monitored with the redox indicator resazurin (1 µg/ml). Sterility was controlled by incubation of medium without bacteria, one well for every plate. Anaerobic cultures were incubated in a Tecan Infinite F200 Pro microplate reader at 37°C for 40 h. OD_600_ was measured at intervals of approximately 15 min in multiple reads per well as recommended by the manufacturer. Anaerobic cultures were shaken immediately before the measurement at 450 rpm for 20 sec. For better comparison with data obtained by measurements with standard spectrophotometers, OD_600_ values were correlated to 1 cm path length by the following formula: OD_600__[transformed]_ = OD_600__[Tecan]_/0.1069. To determine the specific growth rate, the logarithm of the optical density was plotted against time. The slope in the exponential growth phase corresponds to the growth rate (µ). The doubling time (t_d_) was calculated as follows: t_d_ = ln2/µ×60 (min).

**Table 2:** Primers used for generation of deletion mutants, complementing plasmids, and pGEM-T Easy vectors.

Code	Amplified region	Primer	Reference or source
pGEM-*kduI*	*kduI*	GATTATCGGAGGTTGATGTGGA^a^	This work
		CGTTTATGCCCACAACTAGCG^b^	
pGEM-*kduD*	*kduD*	GCTAGTTGTGGGCATAAACG^a^	This work
		GTAAGAAGAATGAATTAACGCGCC^b^	
pGEM-*kduID*	*kduID*	GATTATCGGAGGTTGATGTGGA^a^	This work
		GTAAGAAGAATGAATTAACGCGCC b	
Δ*kduID*	Flanking region of *kduID*	CACTATCGTTTTCTATTTTCACGCTTCACTGATTATCGGAGGTTGATGTGATTCCGGGGATCCGTCGACC^a^	Baba et al. [44]
		GGCAGGGTCATAAAAGTAAGAAGAATGAATTAACGCGCCAGCCAACCGCCTGTAGGCTGGAGCTGCTTCG^b^	
*kduID* control	Up−/downstream of *kduID*	ATATTCGTGATCGACACTGCACTT^a^	This work
		CACGCAGGTGTCAGGTCGGAA^b^	
K2	Kanamycin cassette of pKD13	GCAGTTCATTCAGGGCACCG^b^	Datsenko and Wanner [16]
Kt	Kanamycin cassette of pKD13	CGGCCACAGTCGATGAATCC^a^	Datsenko and Wanner [16]
*kduID*-compl	*kduID*	CGGAATTCGCTCTGCATTTCCTCCTTAC^a^	This work
		TACTGCAGCCATGCGGCAGGGTCATAAA^b^	
pSU19 control	Up−/downstream of multiple-cloning site of pSU19	CCAGGCTTTACACTTTATGC^a^	Rothe et al. [7]
		AGGCTGCGCAACTGTTG^b^	

a forward primer,

b reverse primer.

### Complementation of *E. coli* Mutant Lacking *kduID*

To complement the generated *kduID* deletion mutant, the *kduID* genes, including their corresponding promoters, were amplified from *E. coli* MG1655 with the primers listed in Table 2 and cloned into the low-copy-number plasmid pSU19 as described previously [7], [17]. To characterize aerobic growth, complemented strains containing pSU19 were cultured as described for the deletion mutants but in the presence of chloramphenicol (10 µg/ml).

### Generation and Characterization of Clones Overexpressing KduI and KduD

To investigate a possible role of KduI and KduD in the conversion of galacturonate and glucuronate, both proteins were overexpressed in *E. coli*. Therefore, chromosomal regions of *kduI*, *kduD*, or both genes were amplified from *E. coli* MG1655 by PCR (primers are listed in Table 2), cloned with the pGEM-T Easy Vector System (Figure S3 in the supplemental material), and transformed into *E. coli* JM109 or KRX (Promega Corporation, Madison, USA). Correct insertion was checked by sequencing (Eurofins MWG Operon, Ebersberg, Germany) using plasmid specific primers as described by the manufacturer. Clones with correct insert were precultured aerobically in Terrific Broth (TB) (tryptone [12.0 g/liter], yeast extract [24.0 g/liter], glycerol [4 ml/liter], 89 mM potassium phosphate) plus carbenicillin (50 µg/ml), inoculated at 1% into 10 ml fresh Terrific Broth plus carbenicillin (50 µg/ml), and shaken at 220 rpm and 37°C until an OD_600_ of 0.8–1.0 was reached. Dependent on the orientation of the cloned genes, rhamnose (0.1%) or IPTG (1 mM) were added to induce gene expression. The corresponding cultures were incubated at 220 rpm and 25°C for 16 h. Protein expression was controlled by Laemmli SDS-PAGE [18] (Figure S4 in the supplemental material).

For preparation of cell-free extracts, *E. coli* JM109 pGEM-T, *E. coli* KRX (negative controls), *E. coli* JM109 pGEM-T-*kduID*, *E. coli* KRX pGEM-T-*kduI*, and *E. coli* KRX pGEM-T-*kduD* were grown in 10 ml TB and gene expression was induced as described above. Cells were harvested by centrifugation at 5,000×g and 4°C for 5 min and bacterial pellets were washed twice with 2 ml sodium phosphate buffer (100 mM, pH 7.0) containing a 1∶100-diluted 100× protease inhibitor mix (Roche Diagnostics GmbH, Mannheim, Germany). Cell concentration was adjusted to 2.5×10^10^ cells/ml in a volume of approximately 2 ml. Cells were disrupted with a FastPrep-24 instrument (MP Biomedicals Germany, Eschwege, Germany) using 0.1 mm zirconium-silica beads (Roth, Karlsruhe, Germany) and three 20-s cycles at a speed of 4.0 m/s. Cell disruption was interrupted by 5-min intervals for cooling of the samples on ice. Unbroken cells were removed by centrifugation at 14,000×g and 4°C for 10 min. The protein concentration of cell-free extracts was determined with a Bradford assay (Bio-Rad, Madrid, Spain), using bovine serum albumin as a reference protein. The conversion of hexuronates by cell-free extracts was monitored as follows: 5.4 µl 107 mM galacturonate or 107 mM glucuronate and 2.3 µl 250 mM NADH (10 mM final concentration each) were added to 50 µl cell-free extract on ice. The reaction was started by incubation at 37°C. Samples were taken at 1, 2, 3, 4, and 6 h. The reaction was stopped by addition of 10 µl 100% (w/v) trichloroacetic acid to 50 µl cell-free extract to a final concentration of 17%. Samples were incubated on ice for 30 min, centrifuged at 10,000×g and 4°C for 10 min, supernatants were collected, and stored at –20°C. Galacturonate and glucuronate concentrations were determined enzymatically as described below. Prior to the experiments, it was checked that the trichloroacetic acid present in the samples did not interfere with the enzymatic assay. Specific activities were calculated as follows: the amount of substrate (nmol/ml) was divided by the incubation time (min) and by the protein concentration (mg/ml).

### Determination of Glucuronate and Galacturonate Concentrations

Galacturonate and glucuronate concentrations were determined enzymatically using uronate dehydrogenase from *Agrobacterium tumefaciens* [19]. Plasmid pETATu containing uronate dehydrogenase was kindly provided by K. L. Jones Prather (MIT, Cambridge, USA). Uronate dehydrogenase was expressed in *E. coli* BL21(DE3) after induction with 1 mM IPTG. Protein purification was performed using the ProBond purification system as described by the manufacturer (Invitrogen Corp., Carlsbad, California, USA) and controlled by Laemmli SDS-PAGE [18]. Enzymatic assays were done as described by Moon et al. [19], except that a 96-well plate format was used. Absorbance increase at 340 nm was measured using a Tecan Infinite F200 Pro microplate reader and concentrations were calculated with the help of standard curves.

To measure the intestinal hexuronate concentrations of mice fed the different diets, 200 µl of the liquid phase of small intestinal, caecal, and colonic contents were lyophilized (Martin Christ Gefriertrocknungsanlagen GmbH, Osterode am Harz, Germany). Dried samples were resuspended in 20 µl ddH_2_O and galacturonate and glucuronate concentrations were measured enzymatically in duplicates as described above. For determination of intracellular hexuronate formation during growth of *E. coli* on glucose or lactose, *E. coli* MG1655 were precultured aerobically in 10 ml M9 minimal medium containing 50 mM glucose or 25 mM lactose and inoculated at 2.5% into 20 ml fresh M9 minimal medium containing the same carbon source and shaken aerobically at 220 rpm at 37°C. Samples of 2.5 ml were withdrawn at 2, 3, 4, 6, and at 16 h. Growth was monitored by measuring OD_600_. Cells were harvested by centrifugation at 10,000×g and 4°C for 5 min, washed, resuspended in 250 µl sodium phosphate buffer (100 mM, pH 7.0), and subsequently disrupted in a FastPrep-24 instrument as described above. The protein concentration of cell-free extracts was determined as described above. Cellular proteins were removed by addition of 13.5 µl 100% (w/v) trichloroacetic acid to 180 µl cell-free extract to a final concentration of 7%. Samples were incubated on ice for 30 min, centrifuged at 10,000×g and 4°C for 10 min, and supernatants were transferred to 96-well plates (in duplicates, each 95 µl). Samples were lyophilized, and galacturonate and glucuronate concentrations were measured as described above.

### Statistical Analysis

Statistical analyses were done with GraphPad Prism 5 (GraphPad, La Jolla, USA). Data were tested for normal distribution with the D’Agostino and Pearson omnibus normality test and the Kolmogorow-Smirnow test. All data were non-normally distributed and given as medians and minima versus maxima or median and 25% and 75% percentile. Statistically significant differences were tested by the Kruskal-Wallis one-way analysis of variance and Dunn’s multiple-comparison test or the Mann-Whitney test. SPSS 16.0 (SPSS, Chicago, IL, USA) was used for descriptions of correlations.

## Results

In a previously reported experiment, three groups of gnotobiotic C3H mice monoassociated with *E. coli* K-12 (MG1655) were fed a diet rich in starch, lactose, or casein for 3 weeks (Figure S1 in the supplementary material) [7] and the proteomes of *E. coli* obtained from mice fed the latter diets were compared with those of mice fed the starch diet. This study not only revealed an upregulation of OxyR-dependent proteins in *E. coli* of mice on the lactose-rich diet but also of KduD. Interestingly, on a casein-rich diet, this enzyme and KduI were repressed [7]. Since the role of KduD and KduI in *E. coli* is obscure, the present study aimed to identify the possible role of these proteins in the adaptation of intestinal *E. coli* to the lactose diet.

### Induction of *kduI* and *kduD* Gene Expression by Galacturonate and Glucuronate

To clarify the role of KduD in intestinal *E. coli* of mice fed the lactose diet, expression of the corresponding gene was measured in response to various substrates that have been detected in the mouse intestine [7] using luciferase reporter gene assays. Lactose, glucose, fructose, galactose, casaminoacids, and the diets used in the previous mouse experiment (starch diet, lactose diet, and casein diet; Figure S1 in the supplementary material) were tested for their effect on *kduD* gene expression (Table 3). Galacturonate and glucuronate were included because they have been shown to induce *kduI* and *kduD* gene expression in *Erwinia chrysanthemi* [12].

**Table 3:** Induction of the *kduD* and *kduI* promoter in *E. coli* MG1655 by mouse diets, carbohydrates, casaminoacids, or hexuronates under aerobic and anaerobic growth conditions.

Substrate^b^	Fold-change^a^			
	*kduD*		*kduI*	
	aerobic	anaerobic	aerobic	Anaerobic
Starch diet (1%)	0.5 (0.5∶0.6)	3.4 (2.9∶4.1)	–	–
Lactose diet (1%)	1.0 (0.8∶1.0)	7.7 (5.8∶8.9)	–	–
Casein diet (1%)	0.2 (0.2∶0.3)	2.6 (2.2∶4.0)	–	–
Galactose (50 mM)	**4.0 (1.7∶4.2)** ***^c^***	–	–	–
Fructose (50 mM)	2.0 (1.5∶2.8))	6.1 (4.1∶25)	–	–
Lactose (25 mM)	1.8 (1.2∶2.2)	3.2 (1.0∶7.8)	–	–
Casaminoacids (50 mM)	1.5 (1.2∶2.8)	9.8 (5.0∶15)	–	–
Glucuronate (50 mM)	**3.2 (2.2∶4.9)** ***^c^***	**20 (17∶28)** ***^d^***	**7.0 (6.0∶10)** ***^d^***	**54 (41∶57)** ***^e^***
Galacturonate (50 mM)	**3.3 (2.3∶3.5)** ***^c^***	**9.4 (6.9∶13)** ***^c^***	**11 (5.0∶14)** ***^e^***	**19 (16∶21)** ***^c^***

a Relative luminescence data for *E. coli* MG1655 carrying p*kduDp::luxAB* or p*kduIp::luxAB* are shown. For both aerobic and anaerobic growth conditions, luciferase activity was normalized to values determined for cells grown on glucose (50 mM). Data are expressed as medians and 25% and 75% percentile (aerobic, n = 5–7; anaerobic, n = 4–7).

b Cultures were grown on M9 minimal medium containing pulverized mouse diets or dietary components under aerobic or anaerobic conditions and shaken at 220 and 120 rpm, respectively, at 37°C for 16 h.

c,d Kruskal-Wallis one-way analysis of variance and Dunn’s multiple-comparison test were used for calculations. *^c^*, P<0.05; *^d^*, P<0.01; *^e^*, P<0.001.

No induction of the *kduD* promoter by any of the diets, fructose, lactose, or casaminoacids was observed under aerobic conditions. Only galactose, galacturonate, and glucuronate increased the *kduD* promoter activity 4.0-fold, 3.3-fold, and 3.2-fold, respectively (Table 3). Under anaerobic conditions, galacturonate and glucuronate led to a 9-fold and 20-fold increase in *kduD* expression. Since *E. coli* K-12 is unable to anaerobically grow in galactose-containing minimal medium [20] it was not possible to investigate the effect of galactose on *kduD* expression.

Since KduI was repressed in intestinal *E. coli* of mice fed the casein diet, only substances that induced the *kdud* gene expression were tested for their effect on the *kduI* promoter activity. As observed for *kduD*, the promoter activity of *kduI* increased in the presence of galacturonate and glucuronate (Table 3). *E. coli* cultivated aerobically on galacturonate or glucuronate had 11-fold or 7-fold higher expression levels than cells grown on glucose. Under anaerobic growth conditions, the presence of galacturonate and glucuronate resulted in 19-fold and 54-fold higher expression levels of *kduI*, respectively. Under all experimental conditions tested, *kduI* expression was 2 to 3-fold higher than that of *kduD*. Both promoters displayed higher luciferase activity under anaerobic compared to aerobic growth conditions. Furthermore, under anaerobic conditions, both promoters had a higher activity on glucuronate than on galacturonate (*kduD*, 2.2-fold; *kduI*, 2.8-fold), whereas almost similar values were observed for aerobically grown *E. coli*.

### Repression of Standard Hexuronate Degrading Enzymes (*uxaCA*, *uxaB*, *uxuAB*) by Carbohydrate-induced Osmotic Stress

The induction of *kduI* and *kduD* gene expression by galacturonate and glucuronate suggested that the corresponding proteins are involved in the conversion of these hexuronates. Until now, no evidence exists that *E. coli*’s KduI and KduD, which were upregulated in intestinal *E. coli* of mice fed a lactose-rich diet [7] are involved in galacturonate and glucuronate breakdown. In *E. coli*, galacturonate and glucuronate degradation is normally catalyzed by uronate isomerase (UxaC), altronate oxidoreductase (UxaB) or mannonate oxidoreductase (UxuB), and altronate dehydratase (UxaA) or mannonate dehydratase (UxuA) [21].

Previously, we have demonstrated that high osmolality in the intestines of mice, caused by feeding a lactose-rich diet, leads to an OxyR-dependent induction of stress-related bacterial proteins (AhpF, Dps) [7]. Interestingly, the promoter region of the *E. coli uxuAB* operon contains an OxyR-binding site and *uxuA* expression was shown to be higher in an *oxyR* deletion background [22]. Based on this and the above described observations, we hypothesized that the hexuronate degrading enzymes UxaABC and UxuAB are repressed by the osmotic effect of lactose in an OxyR-dependent manner and that KduI and KduD compensate for the metabolic function of these enzymes under such conditions.

**Figure 1:**
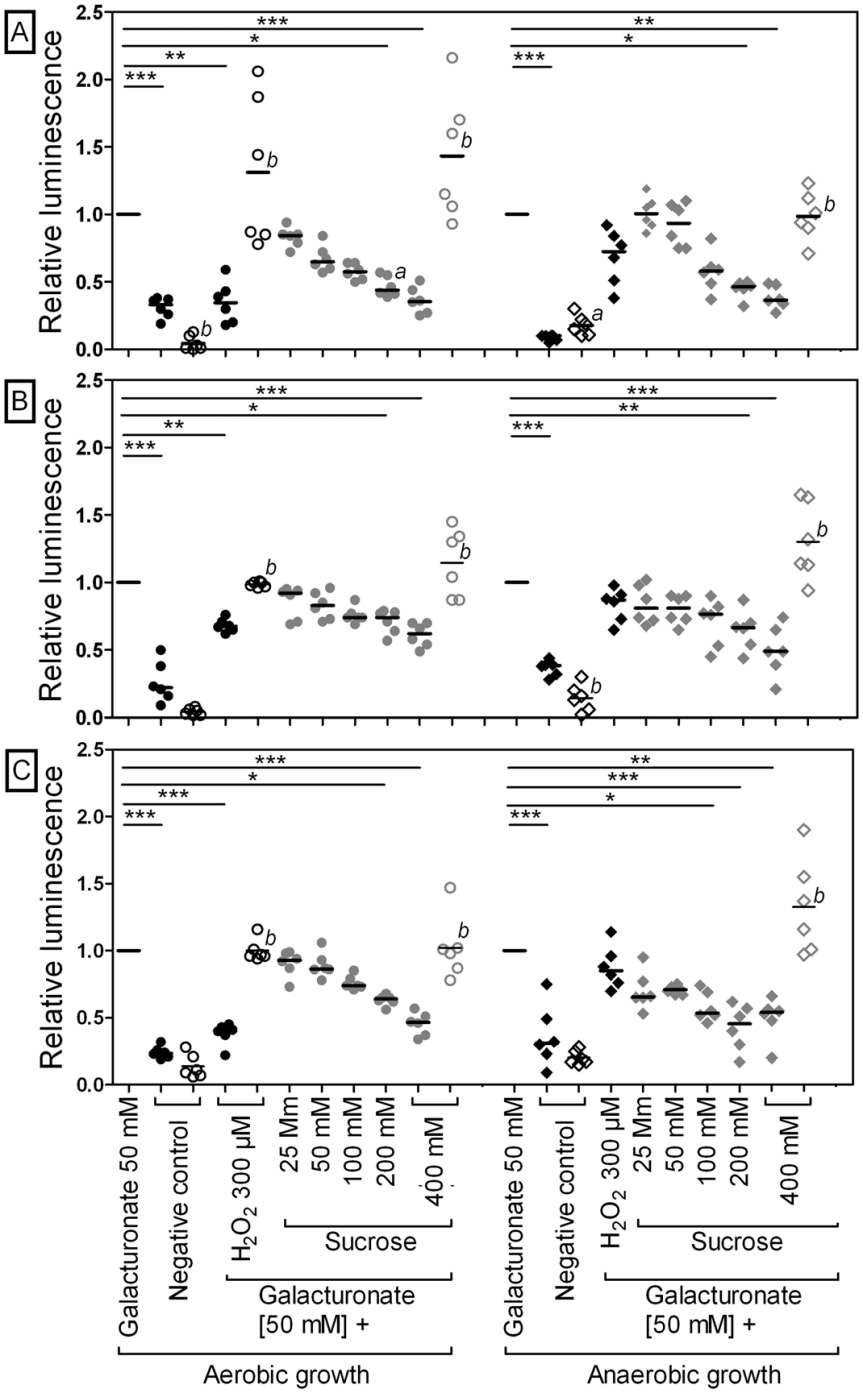
Repression of gene expression of hexuronate degrading enzymes UxaABC and UxuAB by carbohydrate-induced osmotic stress. Activity of the *uxaCA*, *uxaB*, and *uxuAB* promoters in *E. coli* MG1655 (filled symbols) and *E. coli* Δ*oxyR* (open symbols) on M9 minimal medium without substrate (negative control), galacturonate, H_2_O_2_, or sucrose under aerobic (left side, circles) or anaerobic (right side, diamonds) conditions after 90 min of incubation was investigated. Relative luminescence data for *E. coli* MG1655 or *E. coli* Δ*oxyR* carrying p*uxaCAp::luxAB* (A), p*uxaBp::luxAB* (B), or p*uxuABp::luxAB* (C) are shown. Luciferase activity was normalized to values determined for cells grown on 50 mM galacturonate. Data are expressed as medians (n = 6). For values obtained from wild type *E. coli*, the Kruskal-Wallis one-way analysis of variance and Dunn’s multiple-comparison test were used for calculations. *, P<0.05; **, P<0.01; ***, P<0.001. The Mann-Whitney test was applied to compare wild type and mutant strains. *a*, P<0.05; *b*, P<0.01; *c*, P<0.001.

To test this hypothesis, the expression of *uxaCA*, *uxaB*, and *uxuAB* in response to galacturonate and glucuronate with or without osmotic stress was analyzed using luciferase reporter gene assays. Incubation of *E. coli* MG1655 carrying p*uxaCAp::luxAB*, p*uxaBp::luxAB*, or p*uxuABp::luxAB* with galacturonate or glucuronate resulted in an increased promoter activity compared to incubation without hexuronates under both aerobic and anaerobic conditions (aerobic: 3 to 7-fold, anaerobic: 3 to 14-fold) (galacturonate data, Figure 1; glucuronate data, Figure S5 in the supplemental material). Addition of 300 µM H_2_O_2_, a known activator of OxyR, decreased the promoter activity of *uxaCA*, *uxaB*, and *uxuAB* under aerobic but not under anaerobic conditions. To cover a range of osmolalities, non-fermentable sucrose (25–400 mM) was added to galacturonate- or glucuronate-containing minimal medium. The *uxaCA*, *uxaB*, and *uxuAB* promoter activity decreased with increasing sucrose concentrations under both aerobic and anaerobic conditions (400 mM; aerobic, by up to 55%; anaerobic, by up to 60%). The same effect was observed in response to the osmolyte NaCl (400 mM) (data not shown), which supports the notion that osmotic stress is responsible for the observed repression of *uxaCA*, *uxaB*, and *uxuAB*.

To detect a possible correlation between the observed *uxaCA*, *uxaB*, and *uxuAB* promoter activity and the osmolality of the applied media, luciferase activities were plotted against the medium osmolality. Under aerobic and anaerobic conditions, the activity of all promoters negatively correlated with increasing osmolality (Spearman’s rank correlation coefficient: aerobic conditions: *uxaCA*, R^2^ = −0.629 [P≤0.05]; *uxaB*, R^2^ = −0.792 [P≤0.01]; *uxuAB*, R^2^ = −0.818 [P≤0.01]; anaerobic conditions: *uxaCA*, R^2^ = −0.93 [P≤0.01]; *uxaB*, R^2^ = −0.862 [P≤0.01]; *uxuAB*, R^2^ = −0.704 [P≤0.05]).

To obtain further support for the role of OxyR in *uxaCA*, *uxaB,* and *uxuAB* expression, we performed luciferase reporter gene assays in *oxyR* deficient *E. coli*. The *oxyR* deletion strain incubated aerobically with 300 µM H_2_O_2_ had a >2-fold higher expression level of *uxaCA*, *uxaB,* and *uxuAB* than the wild type with a functional *oxyR* (galacturonate data, Figure 1; glucuronate data, Figure S5 in the supplemental material). This is in support of OxyR acting as a repressor on the expression of these genes. In the presence of 400 mM sucrose, the *oxyR* mutant displayed a higher promoter activity than the wild type under both aerobic and anaerobic growth conditions (glucuronate: aerobic, 1.1 to 2.3-fold; anaerobic, 1.6 to 2.5-fold; galacturonate: aerobic, 1.9 to 3.9-fold; anaerobic, 2.4 to 2.6-fold). It can be concluded that carbohydrate-induced osmotic stress led to an OxyR-mediated repression of gene expression of the hexuronate degrading enzymes UxaABC and UxuAB in *E. coli*.

### Osmotic Stress does not Decrease the *kduI* and *kduD* Gene Expression

**Figure 2:**
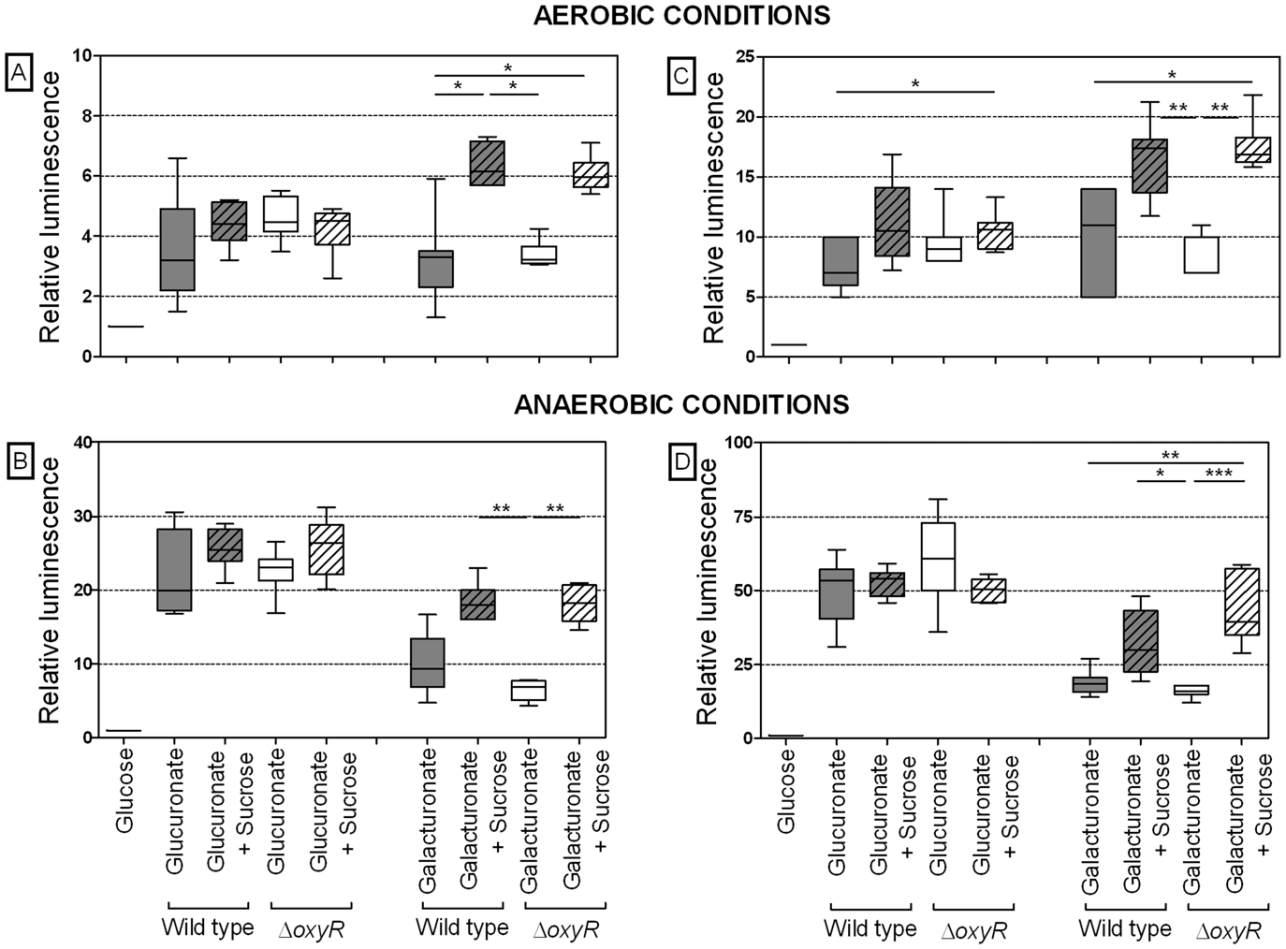
Effect of osmotic stress on hexuronate-induced *kduI* and *kduD* expression in wild type and Δ*oxyR* cells. Expression of *kduD* (A, B) and *kduI* (C, D) in *E. coli* MG1655 (gray bars) and *E. coli* Δ*oxyR* (white bars) in response to galacturonate or glucuronate with and without osmotic stress caused by non-fermentable sucrose under aerobic (A, C) or anaerobic (B, D) conditions after 90 min of incubation was investigated. Cultures were grown on M9 minimal medium containing 50 mM galacturonate or glucuronate in the presence or absence of 400 mM sucrose. Relative luminescence data for *E. coli* MG1655 and *E. coli* Δ*oxyR* carrying p*kduDp::luxAB or* p*kduIp::luxAB* are shown. Luciferase activity was normalized to values determined for cells grown on 50 mM glucose. Data are expressed as medians (aerobic: n = 6–7, anaerobic: n = 4–7). Kruskal-Wallis one-way analysis of variance and Dunn’s multiple-comparison test were used for calculations. *, P<0.05; **, P<0.01.

To investigate the role of KduI and KduD in the conversion of hexuronates, the dependence of the corresponding genes on OxyR and their expression in response to osmotic stress was analyzed. Therefore, luciferase reporter gene assays using wild type and *oxyR*-deficient *E. coli* were performed. Wild type and Δ*oxyR* cells incubated with galacturonate or glucuronate alone did not differ in their *kduI* and *kduD* expression level, neither under aerobic nor anaerobic conditions. Addition of 400 mM sucrose to galacturonate- or glucuronate-containing medium increased the *kduI* and *kduD* expression in both wild type and *oxyR*-deficient *E. coli* (Figure 2). These results indicate that the induction of these genes by hexuronates is OxyR-independent. The finding that osmotic stress did not diminish the expression of *kduI* and *kduD* supports the hypothesis that KduI and KduD may substitute for the function of UxaABC and UxuAB, whose expression is reduced by osmotic stress.

### KduI Facilitates the Conversion of Galacturonate and Glucuronate

**Figure 3:**
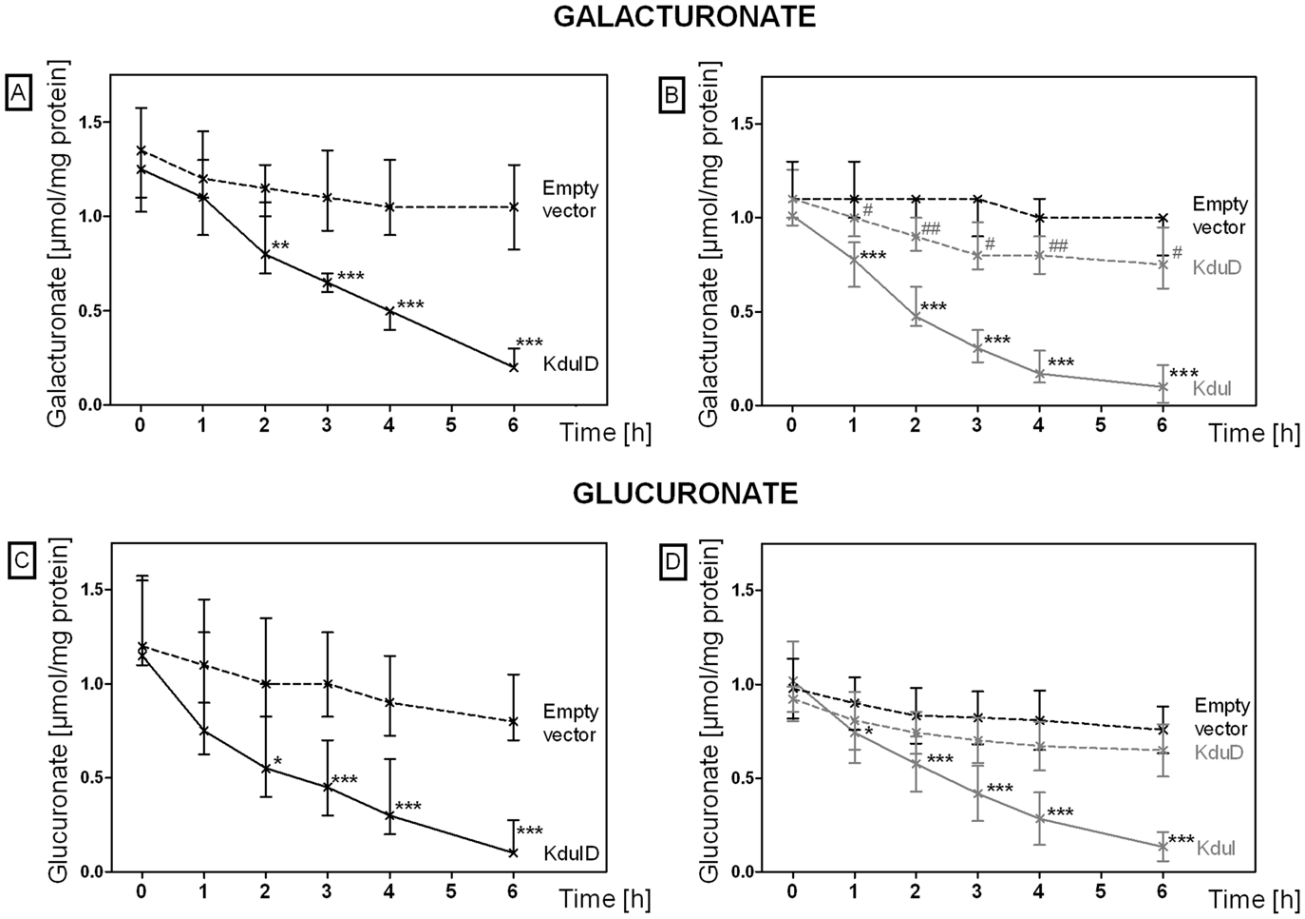
Conversion of galacturonate and glucuronate by cell-free extracts of *E. coli* overexpressing KduI and KduD. Conversion of galacturonate (A, B) and glucuronate (C, D) by cell-free extracts from *E. coli* overexpressing KduI (*E. coli* KRX pGEM-T-*kduI*), KduD (*E. coli* KRX pGEM-T-*kduD*), or both (*E. coli* JM109 pGEM-T-*kduID*) was investigated. *E. coli* strains were selected according to the orientation of the cloned gene: expression in *E. coli* KRX was controlled by the T7-RNA-polymerase and induced by rhamnose (0.1%). Expression in *E. coli* JM109 was controlled by the lacZ-promoter and induced by addition of IPTG (1 mM). *E. coli* containing the empty vector served as a control. The reactions were started by addition of 10 mM galacturonate or glucuronate and incubation at 37°C. Concentration of hexuronates was measured enzymatically. Broken black line, *E. coli* JM109 pGEM-T (A, C), *E. coli* KRX pGEM-T (B, D) (negative controls); black line, *E. coli* JM109 pGEM-T-*kduID* (A, C); gray line, *E. coli* KRX pGEM-T-*kduI*; broken gray line, *E. coli* KRX pGEM-T-*kduD* (B, D). Data are expressed as median and 25% and 75% percentile (n = 11–12). The Mann-Whitney test was used for calculations. *, P<0.05; **, P<0.01; ***, P<0.001.

To gain further support for a role of KduI and KduD in galacturonate and glucuronate conversion under osmotic stress conditions, we tested the ability of *E. coli* to convert these hexuronates. For this purpose, we measured the conversion of galacturonate and glucuronate in cell-free extracts of *E. coli* overexpressing *kduI*, *kduD*, or both. The overexpressed proteins were visualized by SDS-PAGE (Figure S4 in the supplemental material). Extracts from *E. coli* overexpressing both KduI and KduD, or KduI alone converted galacturonate and glucuronate at a higher rate and to a larger extent than cells with an empty vector or cells overexpressing KduD only (Figure 3). This is also reflected by the specific activities of the cell-free extracts (see Table S1 in the supplemental material). These results indicate that KduI facilitates the conversion of galacturonate and glucuronate.

### *kduI* and *kduD* Facilitate the Growth of *E. coli* on Galacturonate and Glucuronate in the Presence of Osmotically Active Sucrose

To evaluate the biological relevance of the observed conversion of hexuronates in the presence of KduI, complementation of *E. coli* mutants lacking *kduID* and *uxaC* with plasmids carrying *kduI* and *kduD* would be the most appropriate approach. However, for unknown reasons we have so far been unsuccessful in generating such mutants. We therefore performed growth experiments with wild type and *kduID*-deficient *E. coli* in galacturonate- or glucuronate-containing (50 mM each) minimal medium under osmotic stress conditions, which repressed the expression of *uxaCA*, *uxaB*, and *uxuAB* (Figure S6 and Figure S7 in the supplementary material). Both strains grew to similar maximal cell densities and had equal doubling times when galacturonate or glucuronate served as energy source (Table 4). The presence of non-fermentable sucrose (200 mM on glucuronate, 400 mM on galacturonate medium), which repressed the expression of *uxaCA*, *uxaB*, and *uxuAB*, led to 1.5-fold longer doubling times of *kduID*-deficient *E. coli* compared to the wild type, whereas the maximal cell densities did not differ. Increasing the sucrose concentration (400 mM on glucuronate, 700 mM on galacturonate medium) resulted in 1.5 to 2-fold longer doubling times as well as reduced maximal cell densities of the mutant compared to wild type *E. coli*. This effect was more evident under aerobic than under anaerobic growth conditions (reduction of OD600: aerobic, by up to 88%; anaerobic, by up to 30%). These results indicate that KduI and KduD facilitate the growth of *E. coli* on galacturonate and glucuronate in the presence of high concentrations of carbohydrates.

**Table 4:** Growth of *E. coli* MG1655 and *E. coli* Δ*kduID* under aerobic and anaerobic conditions a.

	OD_600_ max.		Doubling time t_d_ (min)	
Medium b	*E. coli* MG1655	*E. coli* Δ*kduID*	*E. coli* MG1655	*E. coli* Δ*kduID*
**Aerobic growth conditions**				
Glucuronate	4.8 (4.7∶4.9)	4.8 (4.6∶5.0)	75 (74∶80)	80 (78∶83)*^c^*
Glucuronate, sucrose [200 mM]	4.2 (4.0∶4.5)	3.8 (3.5∶4.0)*^d^*	86 (81∶98)	182 (179∶189)*^d^*
Glucuronate, sucrose [400 mM]	1.4 (1.2∶2.0)	0.3 (0.3∶0.7)*^d^*	141 (128∶156)	270 (221∶326)*^d^*
Galacturonate	5.9 (5.8∶6.1)	5.9 (5.6∶6.1)	75 (71∶76)	73 (72∶103)
Galacturonate, sucrose [400 mM]	4.0 (3.8∶4.3)	4.0 (3.8∶4.3)	114 (109∶120)	252 (200∶278)*^d^*
Galacturonate, sucrose [700 mM]	1.9 (1.0∶2.7)	0.22 (0.14∶0.56)*^d^*	159 (133∶186)	382 (284∶522)*^d^*
**Anaerobic growth conditions**				
Glucuronate	0.71 (0.62∶0.76)	0.7 (0.61∶0.89)	114 (99∶145)	118 (101∶165)
Glucuronate, sucrose [200 mM]	0.43 (0.41∶0.5)	0.35 (0.3∶0.36)*^d^*	202 (171∶225)	281 (241∶453)*^d^*
Glucuronate, sucrose [400 mM]	0.35 (0.35∶0.4)	0.25 (0.21∶0.29)*^d^*	237 (223∶377)	382 (358∶441)*^c^*
Galacturonate	0.76 (0.58∶0.85)	0.69 (0.65∶0.82)	133 (129∶153)	125 (122∶153)
Galacturonate, sucrose [400 mM]	0.36 (0.35∶0.49)	0.34 (0.34∶0.36)	183 (151∶265)	309 (270∶380)*^d^*
Galacturonate, sucrose [700 mM]	0.35 (0.3∶0.37)	0.24 (0.22∶0.28)*^d^*	309 (271∶340)	424 (402∶537)*^d^*

a Data are expressed as medians and minima versus maxima (aerobic, n = 6; anaerobic, n = 5).

b Cultures were incubated in M9 minimal medium containing 50 mM galacturonate or glucuronate with or without sucrose.

c,d Data represent comparisons of the results obtained with *E. coli* MG1655 versus *E. coli* Δ*kduID* that included use of the same medium (Mann-Whitney test; *^c^*, P<0.05; *^d^*, P<0.01).

To demonstrate that the observed effects were indeed KduID-dependent, the deletions causing the observed growth defects were complemented by plasmids containing the corresponding genes and physiologically relevant promoters. Mutants containing these plasmids restored wild type behavior (Figure S8, Table S2 in the supplemental material), confirming that the observed growth inhibition of the deletion mutant in the presence of sucrose was not due to a secondary mutation.

### Galacturonate and Glucuronate are Generated by Growth of *E. coli* on Lactose

The obtained results support the notion that KduI and KduD facilitate the galacturonate and glucuronate conversion under conditions of high osmolality, under which the expression of the regular hexuronate degrading enzymes UxaABC and UxuAB is repressed. Since a lactose-rich diet fed to mice monoassociated with *E. coli* led to an upregulation of KduD [7], the question arose whether galacturonate and glucuronate were available at higher levels in the gut of these mice and whether such hexuronates may originate from dietary lactose. Therefore, the hexuronate concentration was measured in the intestinal contents of mice using an uronate dehydrogenase assay. The concentrations of hexuronates in small intestinal, cecal, and colonic contents were higher in mice fed the lactose diet (104 (30∶185), 28 (11∶51), 14 (10∶44) µg per g [ww]) than in mice fed the starch diet (40 (0∶90), 14 (7∶25), 5 (0∶25) µg per g [ww]) or the casein diet (42 (0∶112), 0 (0∶88), 0 (0∶4) µg per g [ww]). The maximal concentration of 104 µg hexuronates per g [ww] corresponds to 0.48 mM galacturonate or 0.44 mM glucuronate.

**Figure 4:**
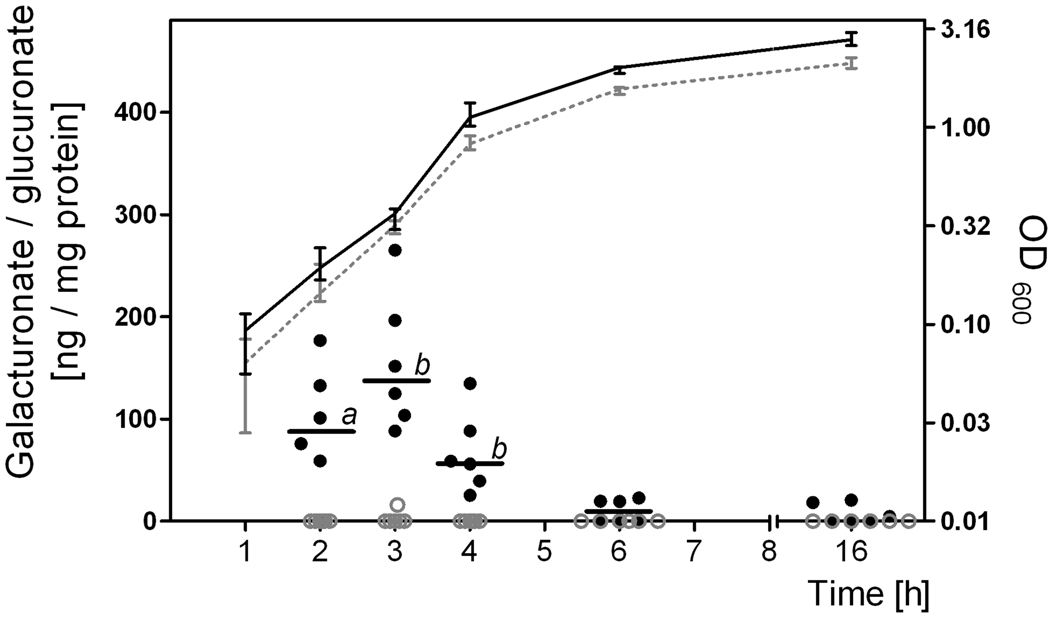
Formation of intracellular galacturonate and glucuronate during growth of *E. coli* on lactose. Intracellular galacturonate and glucuronate concentration was monitored during growth of *E. coli* MG1655 on M9 minimal medium containing 25 mM lactose or 50 mM glucose under aerobic conditions. Cell densities were determined at 600 nm. OD_600_ on glucose medium (dashed gray line), and on lactose medium (black line); intracellular galacturonate concentration on glucose medium (gray dots), and on lactose medium (black dots). Data are expressed as medians (n = 6). The Mann-Whitney test was applied. *a*, P<0.05; *b*, P<0.01.

To test whether hexuronates possibly originate from dietary lactose, *E. coli* was grown in minimal media containing glucose (50 mM) or lactose (25 mM) and the intracellular galacturonate and glucuronate concentration was determined at selected time points. Due to the low cell density of *E. coli* grown anaerobically on minimal medium, this experiment could only be performed under aerobic conditions. Intracellular hexuronate was only detected when *E. coli* was grown on lactose but not when it was grown on glucose (Figure 4). The highest hexuronate concentration was observed during the exponential growth phase (138 ng per mg protein). Based on the assumption that 1 mg protein corresponds to a cytoplasmic volume of 2 µl [23], [24], [25], 138 ng hexuronate per mg protein corresponds to a concentration of 0.32 mM galacturonate or 0.3 mM glucuronate in the bacterial cytoplasm. In the stationary phase, the hexuronate concentrations decreased continuously (2.4 ng per mg protein). The transient formation and disappearance of hexuronates during growth on lactose suggested that these hexuronates served as substrates for *E. coli*. Based on the finding that KduI and KduD facilitated the conversion of galacturonate and glucuronate, it may be concluded that expression of these enzymes is to the advantage of *E. coli* under conditions, in which the typical hexuronate-degrading enzymes are repressed.

## Discussion

We previously reported that a lactose-rich diet fed to gnotobiotic mice not only induced OxyR-dependent stress-response proteins in intestinal *E. coli* but also the poorly characterized protein KduD [7]. This was an unexpected finding because KduD and KduI are involved in pectin degradation in *Erwinia chrysanthemi*. In this organism, gene expression of *kduI* is induced by galacturonate and polygalacturonate [12]. In contrast, *E. coli* is not able to utilize pectin, poly- and oligogalacturonates [13]. This suggests that the induction of KduD in intestinal *E. coli* in response to a lactose-rich diet must have different reasons.

Reporter gene experiments identified galacturonate and glucuronate as inducers of the *kduI* and *kduD* gene expression. A role of KduI in the conversion of galacturonate and glucuronate in *E. coli* was demonstrated by comparing extracts of cells that did or did not overexpress KduI, KduD, or both. However, in which way KduI and/or KduD facilitate the degradation of these hexuronates is unclear. Possible explanations include: i) KduI and KduD stabilize UxaC and/or UxaB or UxuB, ii) they co-induce transporters that facilitate hexuronate uptake, iii) they activate as yet unknown hexuronate degrading pathways, or iv) they are directly involved in galacturonate and glucuronate conversion. Our experiments do no support the first and second explanation, since in the absence of hexuronates, the expression of UxaABC and UxuAB is repressed (Figure 1) [26], [27]. Therefore, stabilization of UxaABC and UxuAB by KduI or KduD cannot take place. Since the KduI- and KduD-dependent hexuronate conversion was determined in cell-free extracts, the observed decrease of galacturonate and glucuronate in these extracts cannot be explained by KduI- and KduD-dependent induction of alternative hexuronate transporters. However, we cannot rule out that KduI activates alternative hexuronate degrading pathways, thereby facilitating the conversion of galacturonate and glucuronate. We consider a catabolic role of KduI and KduD in the utilization of galacturonate and glucuronate the most likely explanation.

**Figure 5:**
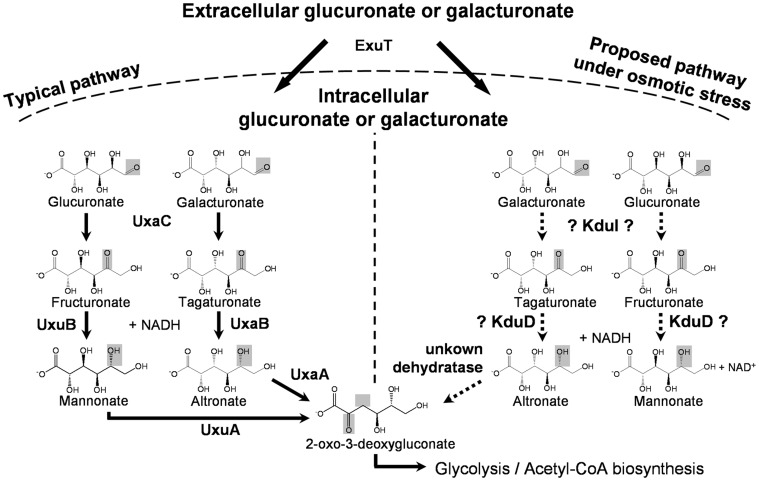
Proposed mechanism of how hexuronates may be converted by KduI and KduD in *E. coli*. In the classical pathway, galacturonate and glucuronate enter the bacterial cell by the aldohexuronate transporter (ExuT) and subsequently undergo isomerization to tagaturonate or fructuronate by uronate isomerase (UxaC). The altronate oxidoreductase (UxaB) or mannonate oxidoreductase (UxuB) catalyze their NADH-dependent reduction to altronate or mannonate, which are further converted by altronate dehydratase (UxaA) or mannonate dehydratase (UxuA) to 2-oxo-3-deoxygluconate. Our results indicate that the 5-keto 4-deoxyuronate isomerase (KduI) and the 2-deoxy-D gluconate 3-dehydrogenase (KduD) may compensate for reduced levels of UxaC, UxaB, and UxuB under osmotic stress conditions. The arrows indicate established, the broken arrows indicate hypothetical reactions.

In *E. coli*, galacturonate and glucuronate enter the cell by the aldohexuronate transporter (ExuT) [28]. In the classical pathway, they subsequently undergo isomerization to tagaturonate or fructuronate by uronate isomerase (UxaC) (Figure 5). In the next step altronate oxidoreductase (UxaB) or mannonate oxidoreductase (UxuB) catalyze the NADH-dependent reduction of tagaturonate or fructuronate to altronate or mannonate, which are further converted by altronate dehydratase (UxaA) or mannonate dehydratase (UxuA) to 2-oxo-3-deoxygluconate [29], [28]. Based on sequence similarity to a 5-keto-4-deoxyuronate isomerase in *E. chrysanthemi*, which catalyzes the conversion of 5-keto-4-deoxyuronate to 2,5-dioxo-3-deoxygluconate, *E. coli*’s KduI was predicted to catalyze the same reaction [12]. Based on this and on the structural similarities of galacturonate and glucuronate with 5-keto-4-deoxyuronate we hypothesize that KduI catalyzes the isomerization of these hexuronates to tagaturonate and fructuronate in *E. coli*. If this assumption is true, KduI may compensate for the function of UxaC under osmotic stress conditions, because the *uxaC* transcription is repressed under such conditions in an OxyR-dependent manner. The OxyR transcriptional regulator not only acts as a sensor for oxidative stress [30] but also as a sensor for osmotic stress [7].

In *E. chrysanthemi*, KduD catalyzes the NADH-dependent reduction of 2,5-dioxo-3-deoxygluconate to 2-oxo-3-deoxygluconate. Since KduD was found to be upregulated in intestinal *E. coli* of mice fed the lactose-rich diet and reporter gene assays revealed that hexuronates induced *kduD* gene expression, this enzyme is possibly involved in catalyzing a later step in hexuronate conversion although overexpression of KduD alone did not decrease the hexuronate concentration in cell-free extracts. Therefore, KduD may compensate for the function of UxaB or UxuB, whose encoding genes, as mentioned above for *uxaC*, were repressed by osmotic stress. Based on the proposed function of KduI, this reaction could not occur in the absence of KduI because the KduD-substrates tagaturonate and fructuronate would be missing (Figure 5). Which protein takes over the function of UxaA or UxuA at high osmolality and subsequently converts altronate or mannonate to 2-oxo-3-deoxygluconate is presently unknown. We therefore postulate the presence of another dehydratase. Nevertheless, the proposed pathway needs to be verified by further experiments and more detailed characterization of KduI and KduD.

**Figure 6:**
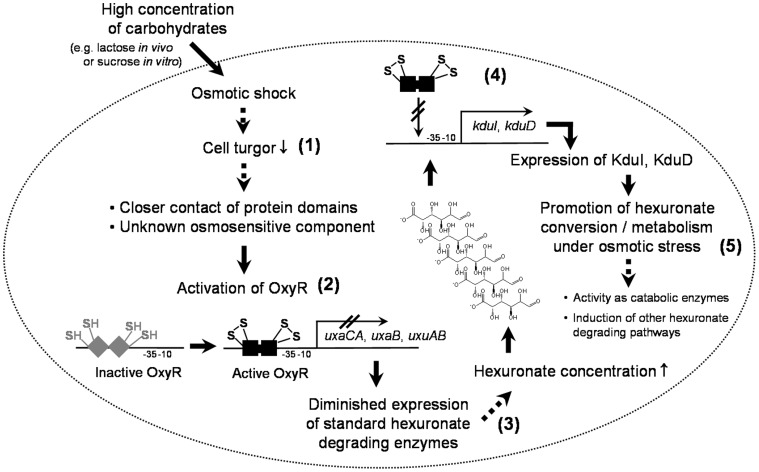
Potential mechanism of how osmotic stress influences the gene expression of hexuronate degrading enzymes. High carbohydrate concentrations caused by lactose *in vivo* or sucrose *in vitro* lead to an osmotic shock (**1**), which is proposed to activate the OxyR transcriptional regulator by a closer contact of normally separated protein domains or an unknown osmosensitive transducing component [7] (**2**). The genes of the standard hexuronate degrading enzymes UxaABC and UxuAB are repressed in an OxyR-dependent manner (**3**) and lead to diminished hexuronate utilization in the presence of osmotic stress. In contrast, expression of *kduI* and *kduD*, which is inducible by galacturonate and glucuronate, is neither osmosensitive nor OxyR-dependent (**4**). Since KduI and KduD facilitate the conversion of galacturonate and glucuronate, these enzymes are proposed to replace UxaC, UxaB, and/or UxuB under conditions of high osmolality (**5**). The arrows indicate experimentally proven reactions; the broken arrows indicate hypothetical functions.

In contrast to *uxaCA*, *uxaB*, and *uxuAB*, our data demonstrate that the expression of *kduI* and *kduD* was independent of OxyR and not diminished under osmotic stress conditions. The crucial role of KduI and KduD in hexuronate metabolism under conditions, in which the standard hexuronate degrading enzymes are repressed, was supported by growth experiments under such conditions (Figure 6). Therefore, it was important to check the origin of galacturonate and glucuronate in the intestine of the gnotobiotic mice. Hexuronates were not present in the diets fed to the mice (Figure S1 in the supplementary material), but they appear as sugar substituents of glycoproteins and glycolipids in the mammalian mucus layer [31], [32] and are therefore expected to occur in intestinal contents of mice. Our results demonstrate that the intestinal hexuronate concentrations were increased in mice fed the lactose diet. However, it is presently not known, whether these hexuronates originate from lactose and, if yes, which pathways are used for their formation. Nevertheless, the transient formation of hexuronates in the cytoplasm of exponentially growing *E. coli* on lactose but not on glucose suggests that the detected cytoplasmic hexuronates may originate from lactose.

To clearly identify possible sources or precursors of the detected intracellular hexuronates, experiments using isotopically labelled lactose would be required. At present, we can only speculate about potential mechanisms by which these lactose-derived hexuronates could be generated from lactose. In *E. coli*, lactose is cleaved to galactose and glucose by ß-galactosidase [33], [34]. While glucose can directly enter glycolysis, galactose is usually converted via the Leloir pathway [35]. The high metabolic rate in the exponential growth phase may result in the accumulation of either intracellular UDP-glucose or galactose as observed for lactic acid-producing bacteria [36], [37]. The uronate dehydrogenase assay used in our study cannot distinguish between galacturonate and glucuronate [19]. Therefore, it has not been possible to clearly identify the source of the detected intracellular hexuronates. Two possibilities are conceivable: 1. Intracellular glucuronate may stem from UDP-glucuronate, which in turn is produced from UDP-glucose as catalyzed by UDP-glucose 6-dehydrogenase (Ugd) [38]. 2. Intracellular galacturonate may be produced by oxidation of galactose. However, such a reaction has not been demonstrated in *E. coli*.

The disappearance of intracellular hexuronates during transition from exponential to stationary growth phase of *E. coli* indicates utilization of these compounds. In the intestine of mice fed a lactose-rich diet and monoassociated with *E. coli*, lactose is readily available in the feeding periods [7], enabling intestinal bacteria to grow exponentially. Exponential growth on lactose would result in the constant formation of intracellular hexuronates, which in turn induce the alternative hexuronate degrading enzymes KduI and KduD.

Export of intracellular glucuronate via the gluconate transporter (GntT) or facilitated diffusion mechanisms [39], [40], [41], [42], [43] may be the reason for the elevated intestinal hexuronate concentrations in the intestines of the gnotobiotic mice fed a lactose-rich diet. However, there would be no benefit for *E. coli* to release a potential energy source. Moreover, the cytoplasmic galacturonate and glucuronate concentrations of *E. coli* grown on lactose-containing minimal medium were similar but not higher than those in the intestinal contents of mice on a lactose-rich diet. Therefore, export of hexuronates is unlikely to happen.

In conclusion, our study suggests that a lactose-rich diet fed to mice monoassociated with *E. coli* leads to an increased *kduI* and *kduD* gene expression in intestinal *E. coli* by galacturonate and glucuronate, which conversion was facilitated by KduI.

## Supporting Information

Figure S1 **Composition of the semi-synthetic diets (% w/w).** Gnotobiotic mice monoassociated with *E. coli* MG1655 were fed either the starch (control) diet, the lactose diet, or the casein diet for 3 weeks.
(PDF)

Figure S2 **Schematic presentation of the regions deleted on the chromosome of** ***E.*** ** ***coli*** **Δ** ***kduID*** **.** The expression or function of genes adjacent to the deleted *kduID* genes (*yqeF* and *areE*) should not be affected by the constructed deletions because promoter and coding regions remained unchanged in the mutants. Black arrows indicate start and termination sites of genes, red arrows indicate deleted sequences
(PDF)

Figure S3 **Schematic presentation of the P-GEM-T constructs carrying** ***kduI*** **,** ***kduD*** **, or both.** Chromosomal regions coding for *kduI*, *kduD*, or both genes including the ribosomal binding sites (RBS) were amplified from *E. coli* MG1655 and cloned into P-GEM-T vectors (A). Black arrows indicate start and termination sites of genes, red arrows indicate chromosomal sequences cloned in pGEM-T vectors. The orientation of genes was validated by sequencing using plasmid specific primers. Gene expression of clones carrying *kduI* and *kduD* (B) was under the control of the lac promoter and therefore inducible by IPTG. Expression of clones carrying *kduI* (C) or *kduD* (D) was controlled by the T7-RNA-polymerase, which is inducible by addition of rhamnose.
(PDF)

Figure S4 **SDS-PAGE of cell-free extracts from** ***E.*** ** ***coli*** **overexpressing KduI or KduD.** Gene expression of clones carrying *kduI* and *kduD* under the control of the lac promoter (*E. coli* JM109 pGEM-T-*kduID*, lane 2) was induced by addition of IPTG; gene expression of clones carrying either *kduD* (*E. coli* KRX pGEM-T-*kduD*, lane 3) or *kduI* (*E. coli* KRX pGEM-T-*kduI*, lane 4) under the control of the T7-RNA-polymerase was induced by incubation of cells with rhamnose. The empty vector served as negative control (*E. coli* JM109 pGEM-T, lane 1; *E. coli* KRX pGEM-T, lane 5). Per lane 10**µg protein were applied. Black arrows indicate the band of the overexpressed proteins.
(PDF)

Figure S5 **Repression of gene expression of hexuronate degrading enzymes UxaABC and UxuAB by carbohydrate-induced osmotic stress.** Activity of the *uxaCA*, *uxaB*, and *uxuAB* promoters in *E. coli* MG1655 (filled symbols) and *E. coli* Δ*oxyR* (open symbols) on M9 minimal medium without substrate (negative control), glucuronate, H_2_O_2_, or sucrose under aerobic (left side, circles) or anaerobic (right side, diamonds) conditions after 90**min of incubation was investigated. Relative luminescence data for *E. coli* MG1655 or *E. coli* Δ*oxyR* carrying p*uxaCAp::luxAB* (A), p*uxaBp::luxAB* (B), or p*uxuABp::luxAB* (C) are shown. Luciferase activity was normalized to values determined for cells grown on 50**mM glucuronate. Data are expressed as medians (n = 6). For values obtained from wild type *E. coli*, the Kruskal-Wallis one-way analysis of variance and Dunn’s multiple-comparison test were used for calculations. *, P<0.05; **P, <0.01; ***, P<0.001. The Mann-Whitney test was applied to compare wild type and mutant strains. *a*, P<0.05; *b*, P<0.01; *c*, P<0.001.
(PDF)

Figure S6 **Diminished growth of** ***E. coli*** **Δ** ***kduID*** **on galacturonate in the presence of carbohydrate-induced osmotic stress.** *E. coli* MG1655 (black line) and *E. coli* Δ*kduID* (blue line) were incubated in M9 minimal medium containing 50 mM galacturonate (A, D), 50 mM galacturonate and 400****mM sucrose (B, E) or 50 mM galacturonate and 700****mM sucrose (C, F). A – C, aerobic conditions, n = 6; D – F, anaerobic conditions, n = 5. Cell densities were determined at 600****nm; data are expressed as medians and minima versus maxima.
(PDF)

Figure S7 **Diminished growth of** ***E. coli*** **Δ** ***kduID*** **on glucuronate in the presence of carbohydrate-induced osmotic stress.** *E. coli* MG1655 (black line) and *E. coli* Δ*kduID* (gray line) were incubated in M9 minimal medium containing 50 mM glucuronate (A, D), 50 mM glucuronate and 200****mM sucrose (B, E) or 50 mM glucuronate and 400****mM sucrose (C, F). A – C, aerobic conditions, n = 6; D – F, anaerobic conditions, n = 5. Cell densities were determined at 600****nm; data are expressed as medians and minima versus maxima.
(PDF)

Figure S8 **Restoration of wild type growth behaviour of** ***E. coli*** **Δ** ***kduID*** **containing complementing plasmids.** *E. coli p*SU19 (black line) and *E. coli* Δ*kduID p*SU19-*kduID* (gray and blue line) were incubated in M9 minimal medium with 50 mM glucuronate (A), 50 mM glucuronate and 400****mM sucrose (B), 50 mM galacturonate (C), or 50****mM galacturonate and 400****mM sucrose (D) under aerobic conditions. Cell densities were determined at 600****nm; data are expressed as medians and minima versus maxima (n = 6).
(PDF)

Table S1 **Specific activity of KduI and KduD, calculated for hexuronate concentrations observed after incubation of cell-free extracts of** ***E. coli*** **clones overexpressing** ***kduI*** **,** ***kduD*** **, or both genes with 10** ****** **mM galacturonate or glucuronate over 6** ****** **h at 37°C.**
(PDF)

Table S2 **Growth of** ***E. coli*** **MG1655 pSU19 and** ***E. coli*** **Δ** ***kduID*** **pSU19-** ***kduID*** **under aerobic conditions: Complementation of** ***E. coli*** **Δ** ***kduID*** **with plasmids containing the corresponding genes including physiologically relevant promoters restored wild type like behavior.**
(PDF)
