# Supplementary material for: Novel Insights into E. coli’s Hexuronate Metabolism: KduI Facilitates the Conversion of Galacturonate and Glucuronate under Osmotic Stress Conditions
Source: PLoS One. 2013 Feb 21;8(2):e56906. doi: 10.1371/journal.pone.0056906 (PMC3578941; doi:10.1371/journal.pone.0056906)
Supplement: Figure S1 — Composition of the semi-synthetic diets (% w/w). Gnotobiotic mice monoassociated with E. coli MG1655 were fed either the starch (control) diet, the lactose diet, or the casein diet for 3 weeks. (PDF) [file pone.0056906.s001.pdf]

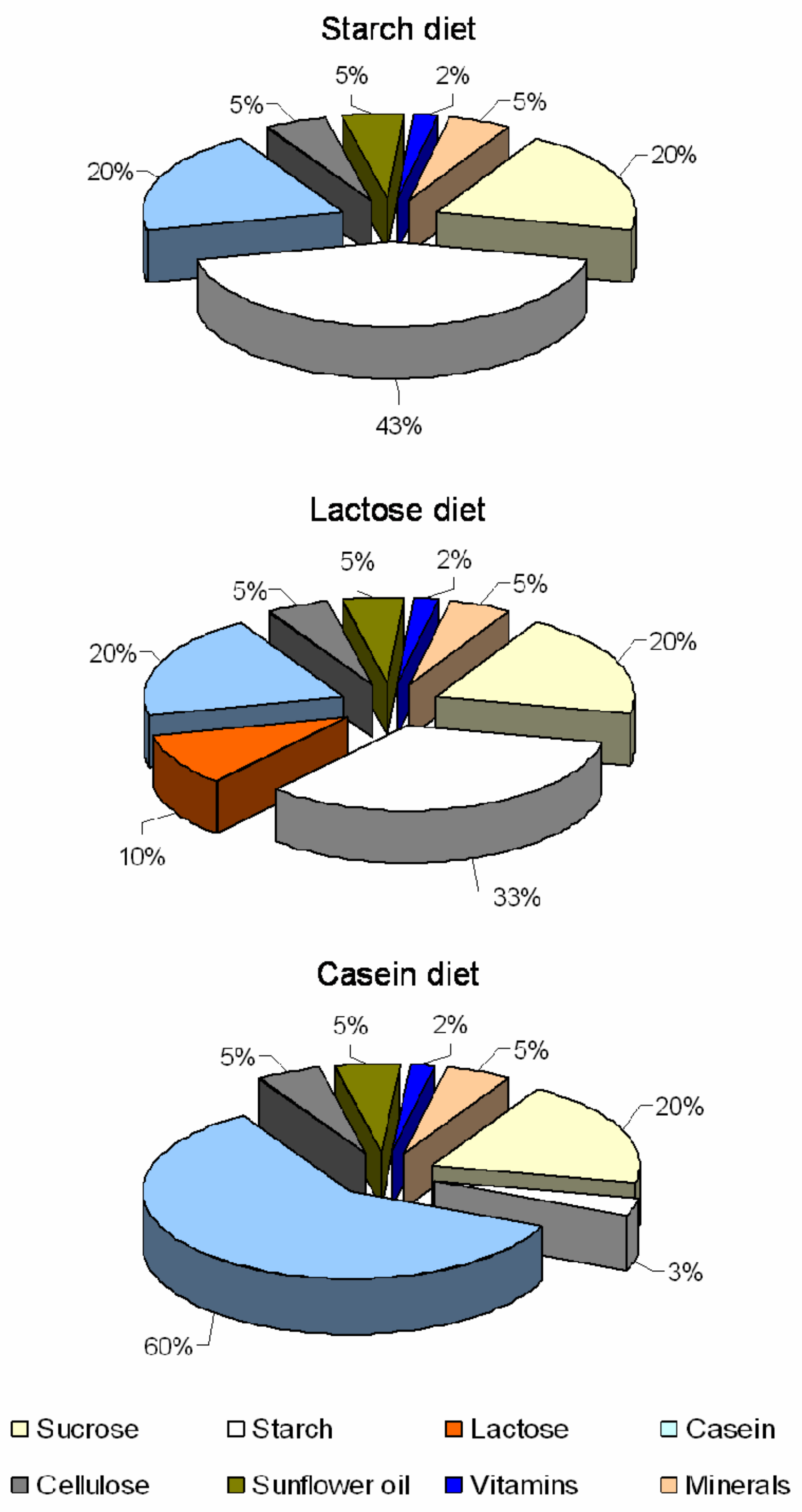


**Figure S1. Composition of the semi-synthetic diets (% w/w).** Gnotobiotic mice monoassociated with *E. coli* MG1655 were fed either the starch (control) diet, the lactose diet, or the casein diet for 3 weeks.
