# Supplementary material for: Novel Insights into E. coli’s Hexuronate Metabolism: KduI Facilitates the Conversion of Galacturonate and Glucuronate under Osmotic Stress Conditions
Source: PLoS One. 2013 Feb 21;8(2):e56906. doi: 10.1371/journal.pone.0056906 (PMC3578941; doi:10.1371/journal.pone.0056906)
Supplement: Figure S2 — Schematic presentation of the regions deleted on the chromosome of E. coli Δ kduID . The expression or function of genes adjacent to the deleted kduID genes (yqeF and areE) should not be affected by the constructed deletions because promoter and coding regions remained unchanged in the mutants. Black arrows indicate start and termination sites of genes, red arrows indicate deleted sequences (PDF) [file pone.0056906.s002.pdf]

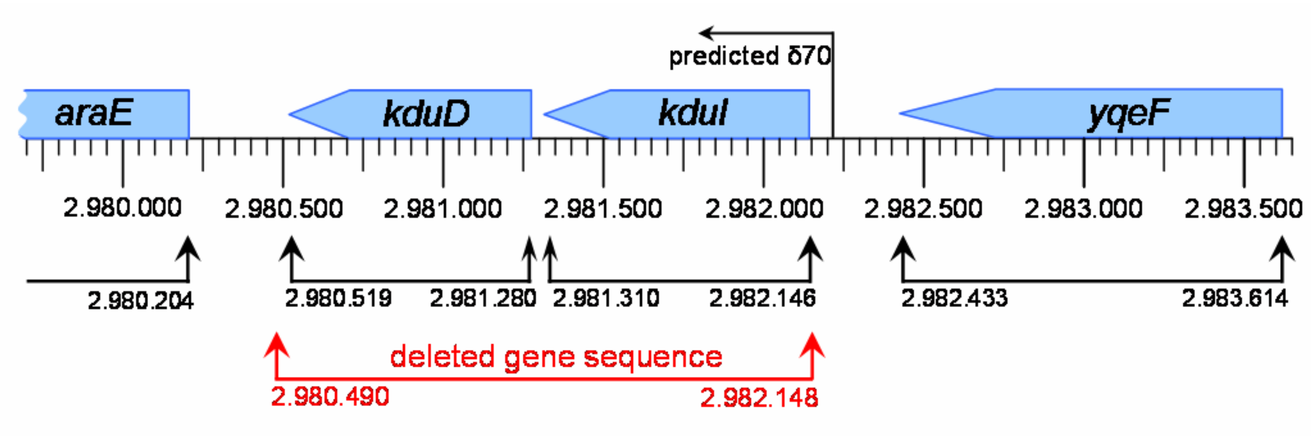

Figure S2. Schematic presentation of the regions deleted on the chromosome of *E. coli* Δ*kduID*. The expression or function of genes adjacent to the deleted *kduID* genes (*yqeF* and *areE*) should not be affected by the constructed deletions because promoter and coding regions remained unchanged in the mutants. Black arrows indicate start and termination sites of genes, red arrows indicate deleted sequences.
