# Supplementary material for: Novel Insights into E. coli’s Hexuronate Metabolism: KduI Facilitates the Conversion of Galacturonate and Glucuronate under Osmotic Stress Conditions
Source: PLoS One. 2013 Feb 21;8(2):e56906. doi: 10.1371/journal.pone.0056906 (PMC3578941; doi:10.1371/journal.pone.0056906)
Supplement: Figure S3 — Schematic presentation of the P-GEM-T constructs carrying kduI , kduD , or both. Chromosomal regions coding for kduI, kduD, or both genes including the ribosomal binding sites (RBS) were amplified from E. coli MG1655 and cloned into P-GEM-T vectors (A). Black arrows indicate start and termination sites of genes, red arrows indicate chromosomal sequences cloned in pGEM-T vectors. The orientation of genes was validated by sequencing using plasmid specific primers. Gene expression of clones carrying kduI and kduD (B) was under the control of the lac promoter and therefore inducible by IPTG. Expression of clones carrying kduI (C) or kduD (D) was controlled by the T7-RNA-polymerase, which is inducible by addition of rhamnose. (PDF) [file pone.0056906.s003.pdf]

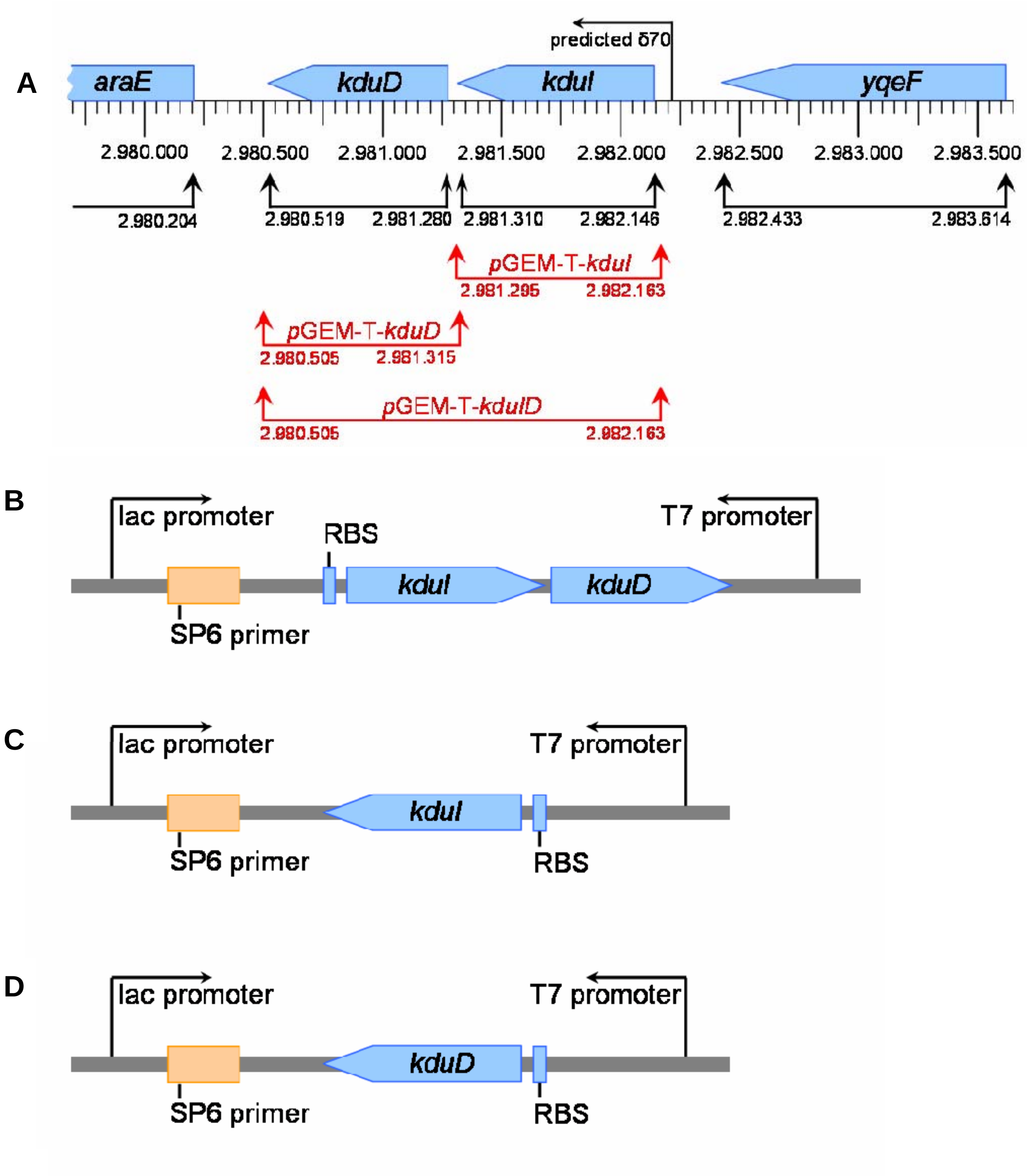

Figure S3. Schematic presentation of the P-GEM-T constructs carrying *kduI*, *kduD*, or both. Chromosomal regions coding for *kduI*, *kduD*, or both genes including the ribosomal binding sites (RBS) were amplified from *E. coli* MG1655 and cloned into P-GEM-T vectors (A). Black arrows indicate start and termination sites of genes, red arrows indicate chromosomal sequences cloned in pGEM-T vectors. The orientation of genes was validated by sequencing using plasmid specific primers. Gene expression of clones carrying *kduI* and *kduD* (B) was under the control of the lac promoter and therefore inducible by IPTG. Expression of clones carrying *kduI* (C) or *kduD* (D) was controlled by the T7-RNA-polymerase, which is inducible by addition of rhamnose.
