# Supplementary material for: Novel Insights into E. coli’s Hexuronate Metabolism: KduI Facilitates the Conversion of Galacturonate and Glucuronate under Osmotic Stress Conditions
Source: PLoS One. 2013 Feb 21;8(2):e56906. doi: 10.1371/journal.pone.0056906 (PMC3578941; doi:10.1371/journal.pone.0056906)
Supplement: Figure S4 — SDS-PAGE of cell-free extracts from E. coli overexpressing KduI or KduD. Gene expression of clones carrying kduI and kduD under the control of the lac promoter (E. coli JM109 pGEM-T-kduID, lane 2) was induced by addition of IPTG; gene expression of clones carrying either kduD (E. coli KRX pGEM-T-kduD, lane 3) or kduI (E. coli KRX pGEM-T-kduI, lane 4) under the control of the T7-RNA-polymerase was induced by incubation of cells with rhamnose. The empty vector served as negative control (E. coli JM109 pGEM-T, lane 1; E. coli KRX pGEM-T, lane 5). Per lane 10µg protein were applied. Black arrows indicate the band of the overexpressed proteins. (PDF) [file pone.0056906.s004.pdf]

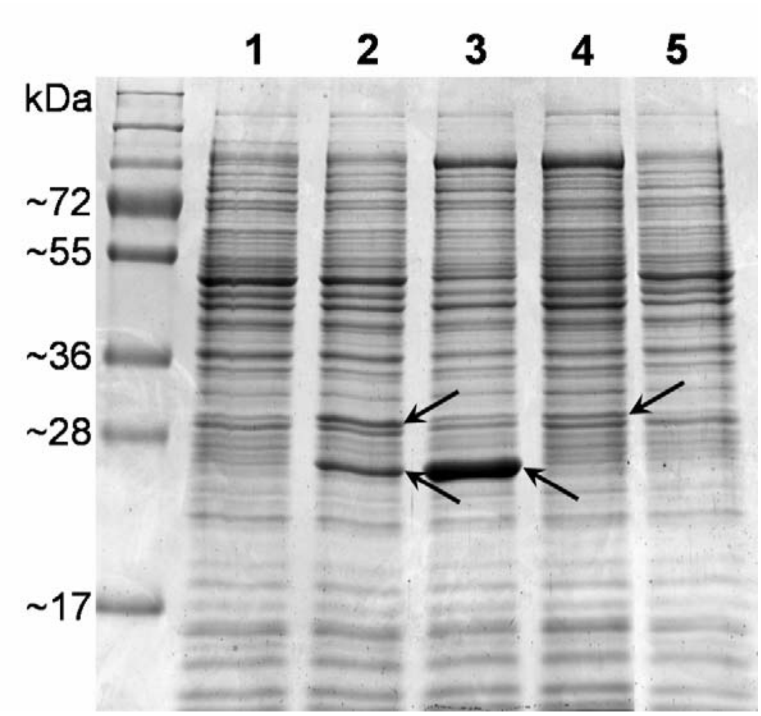

Figure S4. SDS-PAGE of cell-free extracts from *E. coli* overexpressing KduI or KduD. Gene expression of clones carrying *kduI* and *kduD* under the control of the lac promoter (*E. coli* JM109 pGEM-T-*kduID*, lane 2) was induced by addition of IPTG; gene expression of clones carrying either *kduD* (*E. coli* KRX pGEM-T-*kduD*, lane 3) or *kduI* (*E. coli* KRX pGEM-T-*kduI*, lane 4) under the control of the T7-RNA-polymerase was induced by incubation of cells with rhamnose. The empty vector served as negative control (*E. coli* JM109 pGEM-T, lane 1; *E. coli* KRX pGEM-T, lane 5). Per lane 10 µg protein were applied. Black arrows indicate the band of the overexpressed proteins.
