# Supplementary material for: Novel Insights into E. coli’s Hexuronate Metabolism: KduI Facilitates the Conversion of Galacturonate and Glucuronate under Osmotic Stress Conditions
Source: PLoS One. 2013 Feb 21;8(2):e56906. doi: 10.1371/journal.pone.0056906 (PMC3578941; doi:10.1371/journal.pone.0056906)
Supplement: Figure S5 — Repression of gene expression of hexuronate degrading enzymes UxaABC and UxuAB by carbohydrate-induced osmotic stress. Activity of the uxaCA, uxaB, and uxuAB promoters in E. coli MG1655 (filled symbols) and E. coli ΔoxyR (open symbols) on M9 minimal medium without substrate (negative control), glucuronate, H2O2, or sucrose under aerobic (left side, circles) or anaerobic (right side, diamonds) conditions after 90min of incubation was investigated. Relative luminescence data for E. coli MG1655 or E. coli ΔoxyR carrying puxaCAp::luxAB (A), puxaBp::luxAB (B), or puxuABp::luxAB (C) are shown. Luciferase activity was normalized to values determined for cells grown on 50mM glucuronate. Data are expressed as medians (n = 6). For values obtained from wild type E. coli, the Kruskal-Wallis one-way analysis of variance and Dunn’s multiple-comparison test were used for calculations. *, P<0.05; **P, <0.01; ***, P<0.001. The Mann-Whitney test was applied to compare wild type and mutant strains. a, P<0.05; b, P<0.01; c, P<0.001. (PDF) [file pone.0056906.s005.pdf]

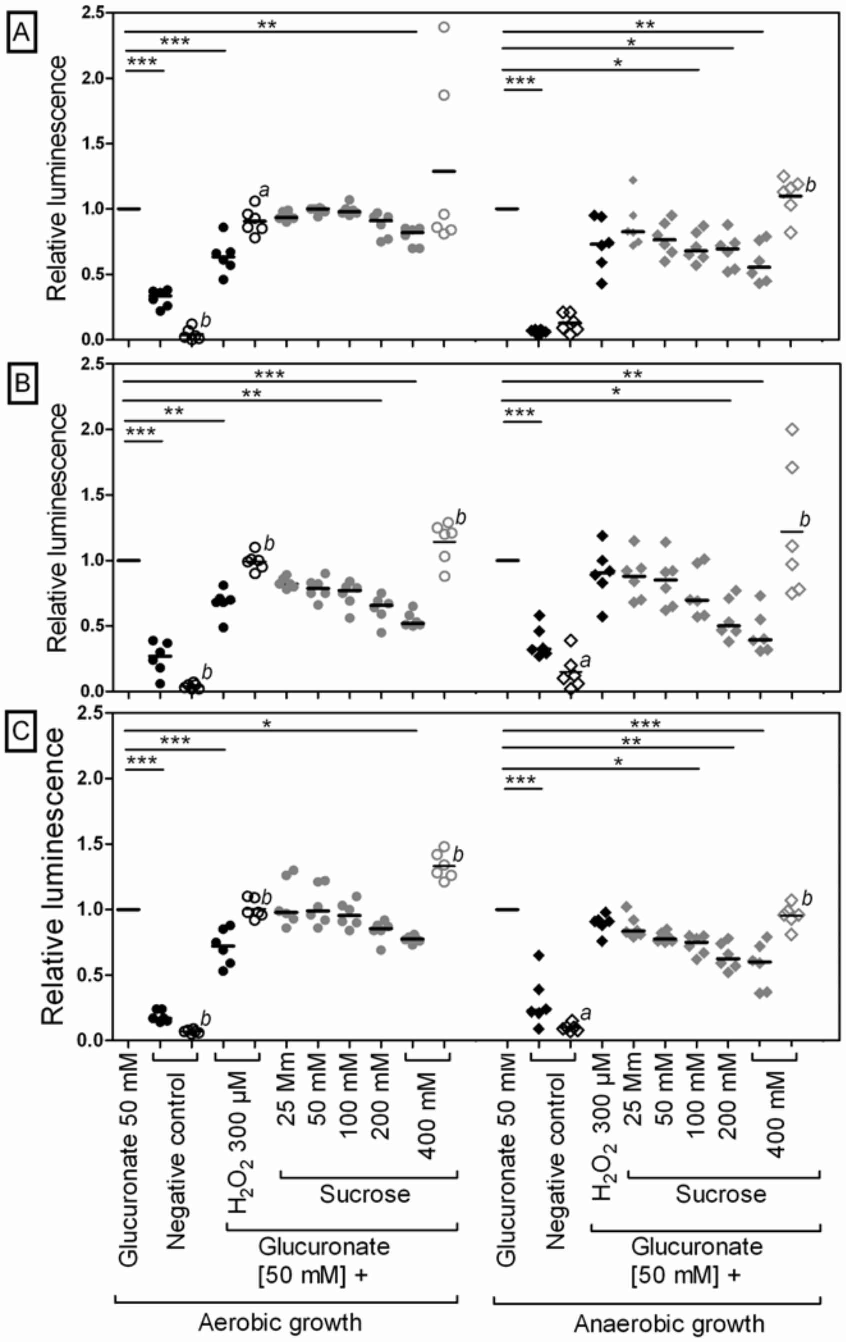


**Figure S5. Repression of gene expression of hexuronate degrading enzymes UxaABC and UxuAB by carbohydrate-induced osmotic stress.** Activity of the *uxaCA*, *uxaB*, and *uxuAB* promoters in *E. coli* MG1655 (filled symbols) and *E. coli* Δ*oxyR* (open symbols) on M9 minimal medium without substrate (negative control), glucuronate, $H_2O_2$, or sucrose under aerobic (left side, circles) or anaerobic (right side, diamonds) conditions after 90 min of incubation was investigated. Relative luminescence data for *E. coli* MG1655 or *E. coli* Δ*oxyR* carrying p*uxaCAp::luxAB* (A), p*uxaBp::luxAB* (B), or p*uxuABp::luxAB* (C) are shown. Luciferase activity was normalized to values determined for cells grown on 50 mM glucuronate. Data are expressed as medians (n = 6). For values obtained from wild type *E. coli*, the Kruskal-Wallis one-way analysis of variance and Dunn's multiple-comparison test were used for calculations. *, P < 0.05; ** P, < 0.01; ***, P < 0.001. The Mann-Whitney test was applied to compare wild type and mutant strains. *a*, P < 0.05; *b*, P < 0.01; *c*, P < 0.001.
