# Supplementary material for: Novel Insights into E. coli’s Hexuronate Metabolism: KduI Facilitates the Conversion of Galacturonate and Glucuronate under Osmotic Stress Conditions
Source: PLoS One. 2013 Feb 21;8(2):e56906. doi: 10.1371/journal.pone.0056906 (PMC3578941; doi:10.1371/journal.pone.0056906)
Supplement: Figure S6 — Diminished growth of E. coli Δ kduID on galacturonate in the presence of carbohydrate-induced osmotic stress. E. coli MG1655 (black line) and E. coli ΔkduID (blue line) were incubated in M9 minimal medium containing 50 mM galacturonate (A, D), 50 mM galacturonate and 400mM sucrose (B, E) or 50 mM galacturonate and 700mM sucrose (C, F). A – C, aerobic conditions, n = 6; D – F, anaerobic conditions, n = 5. Cell densities were determined at 600nm; data are expressed as medians and minima versus maxima. (PDF) [file pone.0056906.s006.pdf]

A

B

C

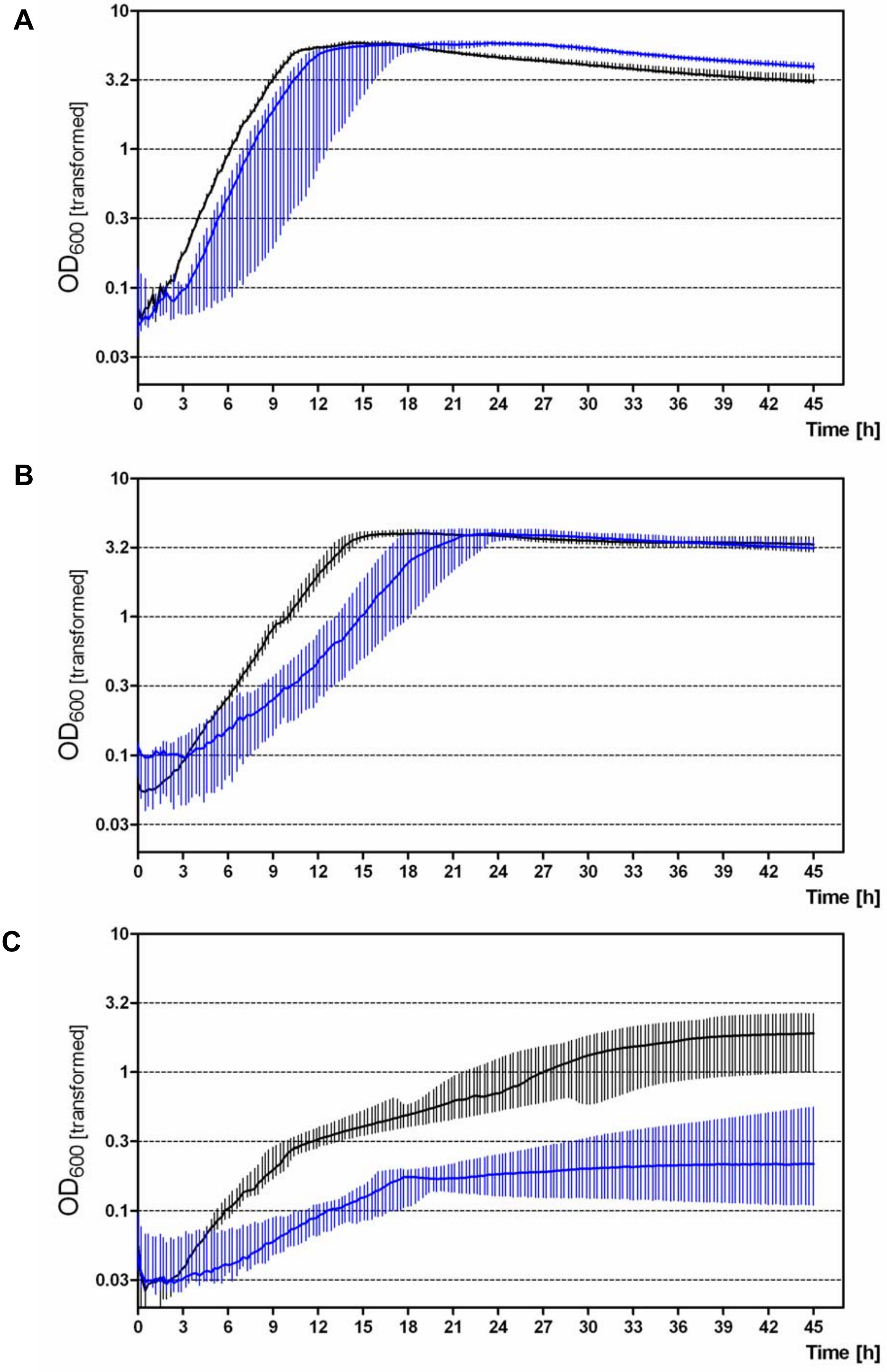

10
3.2
1
0.3
0.1
0.03
OD600 [transformed]
0 3 6 9 12 15 18 21 24 27 30 33 36 39 42 45
Time [h]

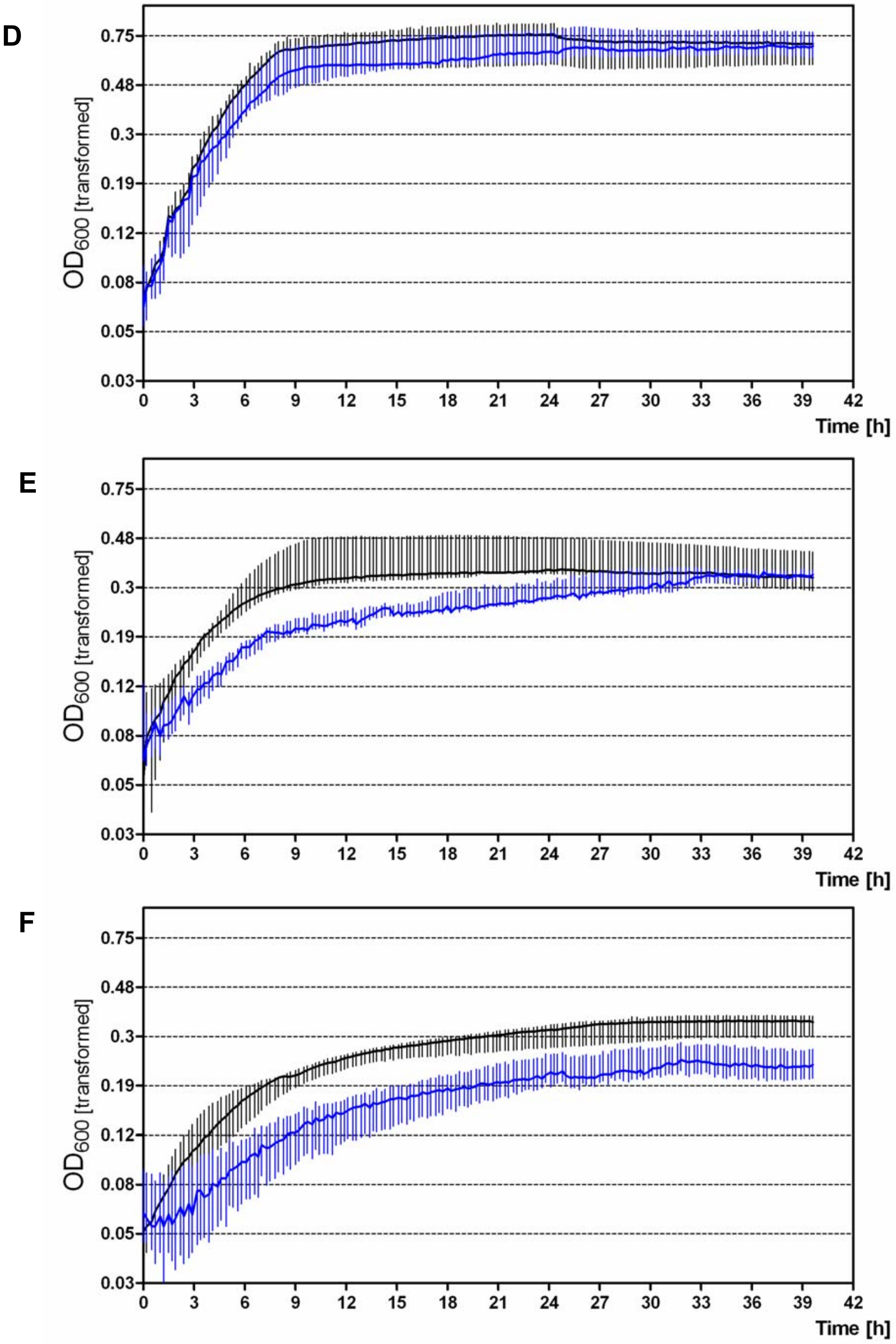

Figure S6. Diminished growth of *E. coli* Δ*kduID* on galacturonate in the presence of carbohydrate-induced osmotic stress. *E. coli* MG1655 (black line) and *E. coli* Δ*kduID* (blue line) were incubated in M9 minimal medium containing 50mM galacturonate (A, D), 50mM galacturonate and 400 mM sucrose (B, E) or 50mM galacturonate and 700 mM sucrose (C, F). A – C, aerobic conditions, n = 6; D – F, anaerobic conditions, n = 5. Cell densities were determined at 600 nm; data are expressed as medians and minima versus maxima.
