# Supplementary material for: Novel Insights into E. coli’s Hexuronate Metabolism: KduI Facilitates the Conversion of Galacturonate and Glucuronate under Osmotic Stress Conditions
Source: PLoS One. 2013 Feb 21;8(2):e56906. doi: 10.1371/journal.pone.0056906 (PMC3578941; doi:10.1371/journal.pone.0056906)
Supplement: Figure S7 — Diminished growth of E. coli Δ kduID on glucuronate in the presence of carbohydrate-induced osmotic stress. E. coli MG1655 (black line) and E. coli ΔkduID (gray line) were incubated in M9 minimal medium containing 50 mM glucuronate (A, D), 50 mM glucuronate and 200mM sucrose (B, E) or 50 mM glucuronate and 400mM sucrose (C, F). A – C, aerobic conditions, n = 6; D – F, anaerobic conditions, n = 5. Cell densities were determined at 600nm; data are expressed as medians and minima versus maxima. (PDF) [file pone.0056906.s007.pdf]

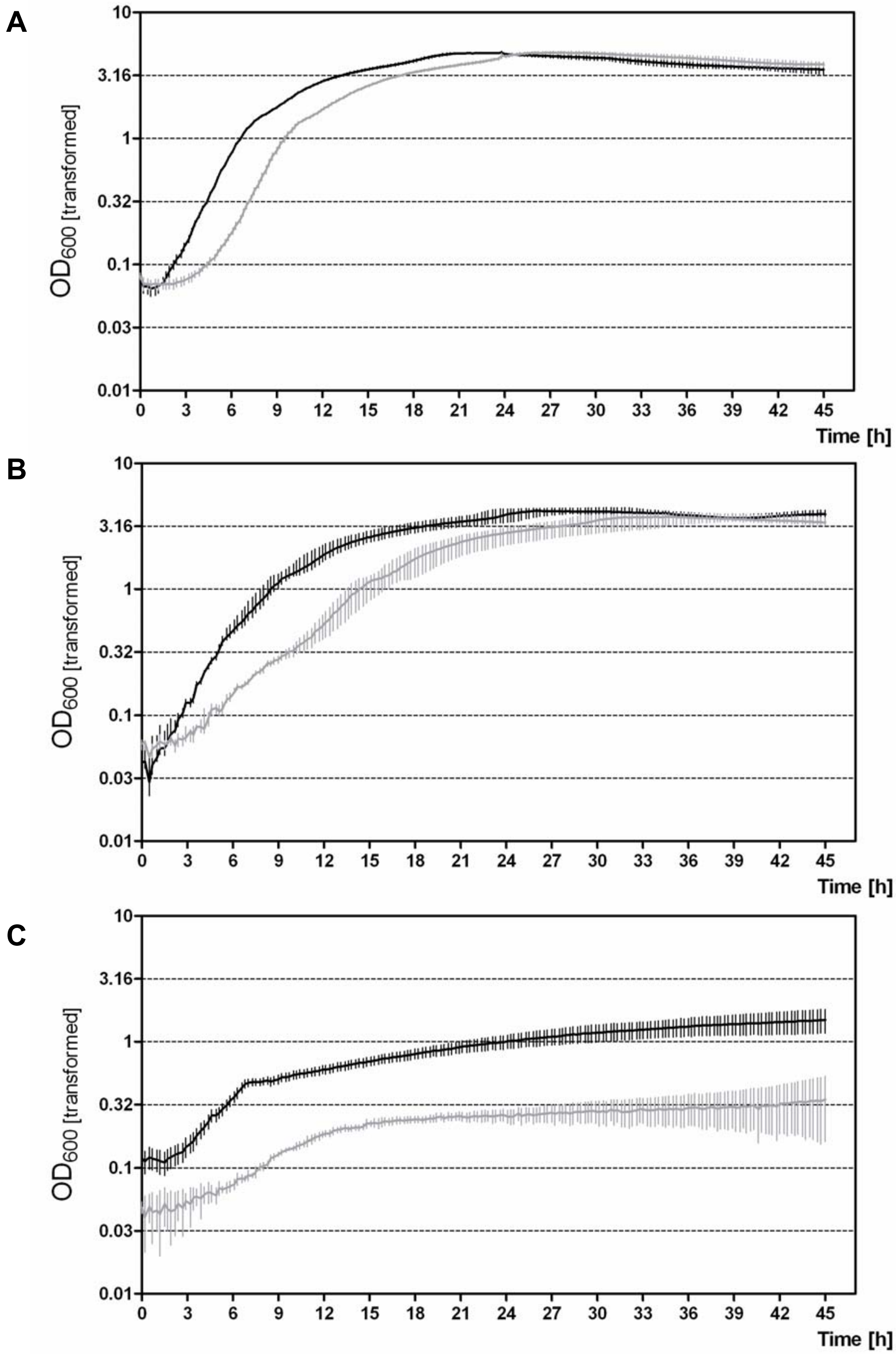

A
B
C
OD600 [transformed]
10
3.16
1
0.32
0.1
0.03
0.01
0 3 6 9 12 15 18 21 24 27 30 33 36 39 42 45
Time [h]

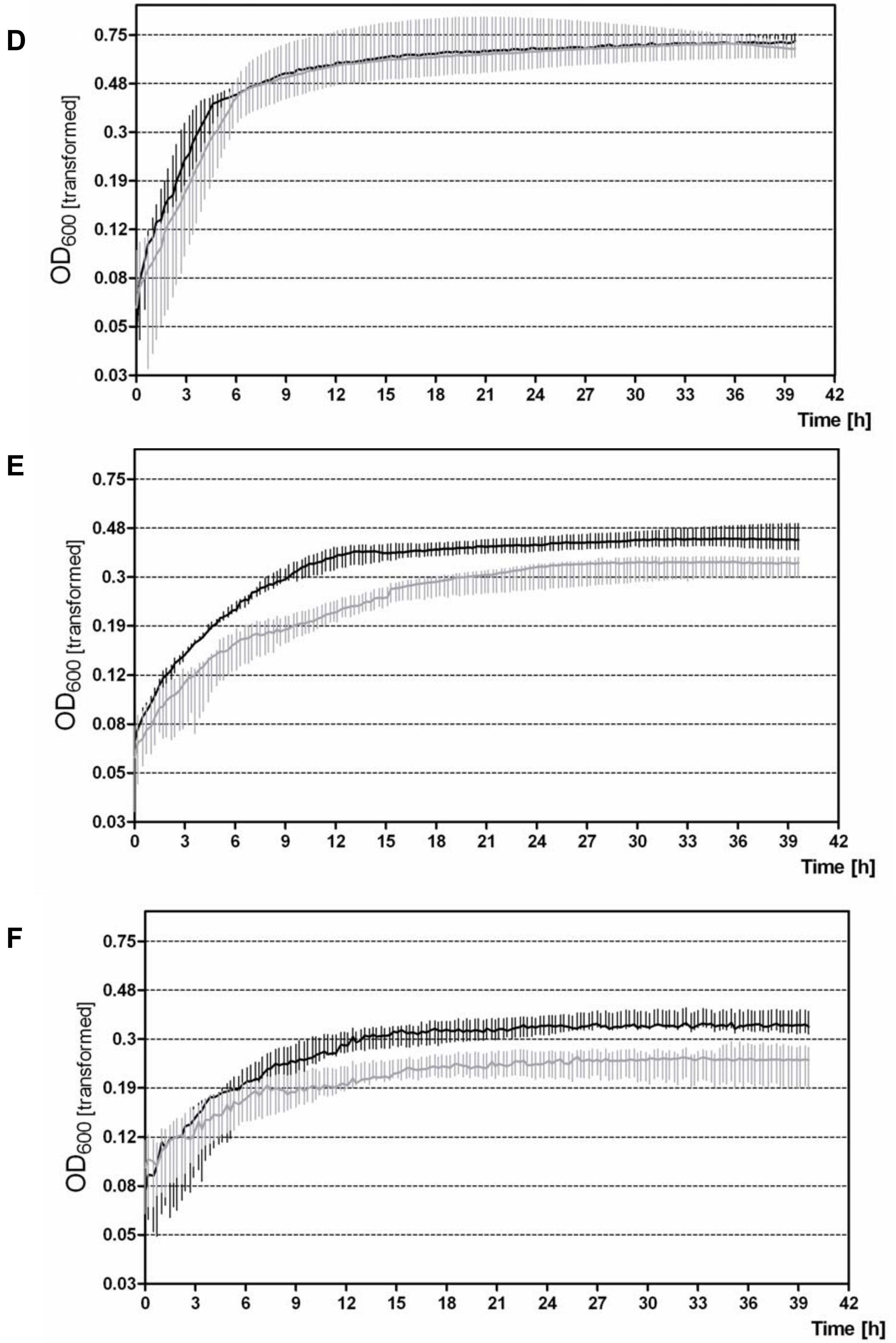

Figure S7. Diminished growth of *E. coli* Δ*kduID* on glucuronate in the presence of carbohydrate-induced osmotic stress *E. coli* MG1655 (black line) and *E. coli* Δ*kduID* (gray line) were incubated in M9 minimal medium containing 50mM glucuronate (A, D), 50mM glucuronate and 200 mM sucrose (B, E) or 50mM glucuronate and 400 mM sucrose (C, F). A – C, aerobic conditions, n = 6; D – F, anaerobic conditions, n = 5. Cell densities were determined at 600 nm; data are expressed as medians and minima versus maxima.
