# Supplementary material for: Novel Insights into E. coli’s Hexuronate Metabolism: KduI Facilitates the Conversion of Galacturonate and Glucuronate under Osmotic Stress Conditions
Source: PLoS One. 2013 Feb 21;8(2):e56906. doi: 10.1371/journal.pone.0056906 (PMC3578941; doi:10.1371/journal.pone.0056906)
Supplement: Figure S8 — Restoration of wild type growth behaviour of E. coli Δ kduID containing complementing plasmids. E. coli pSU19 (black line) and E. coli ΔkduID pSU19-kduID (gray and blue line) were incubated in M9 minimal medium with 50 mM glucuronate (A), 50 mM glucuronate and 400mM sucrose (B), 50 mM galacturonate (C), or 50mM galacturonate and 400mM sucrose (D) under aerobic conditions. Cell densities were determined at 600nm; data are expressed as medians and minima versus maxima (n = 6). (PDF) [file pone.0056906.s008.pdf]

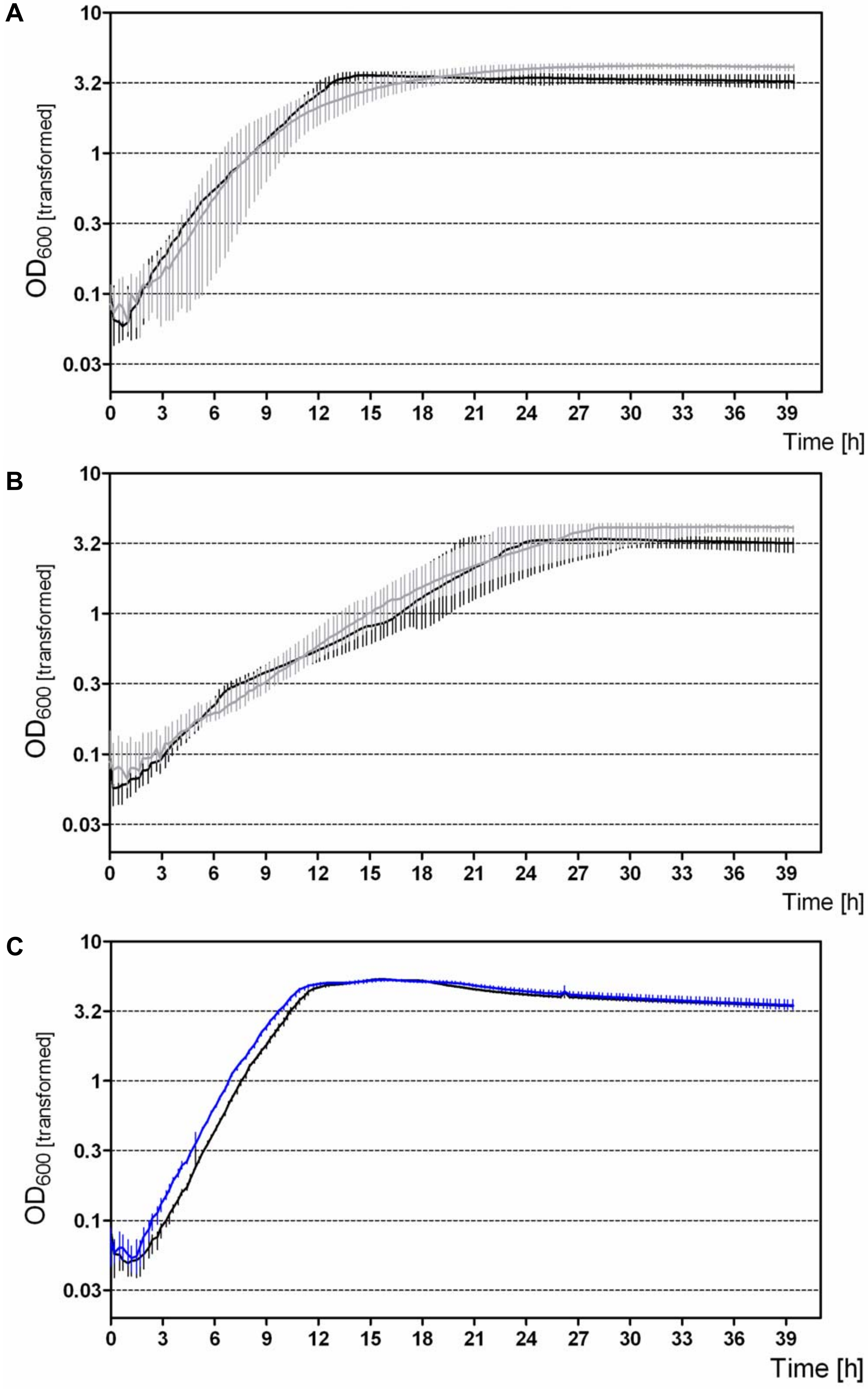

A
B
C
OD600 [transformed]
10
3.2
1
0.3
0.1
0.03
0
3
6
9
12
15
18
21
24
27
30
33
36
39
Time [h]

D

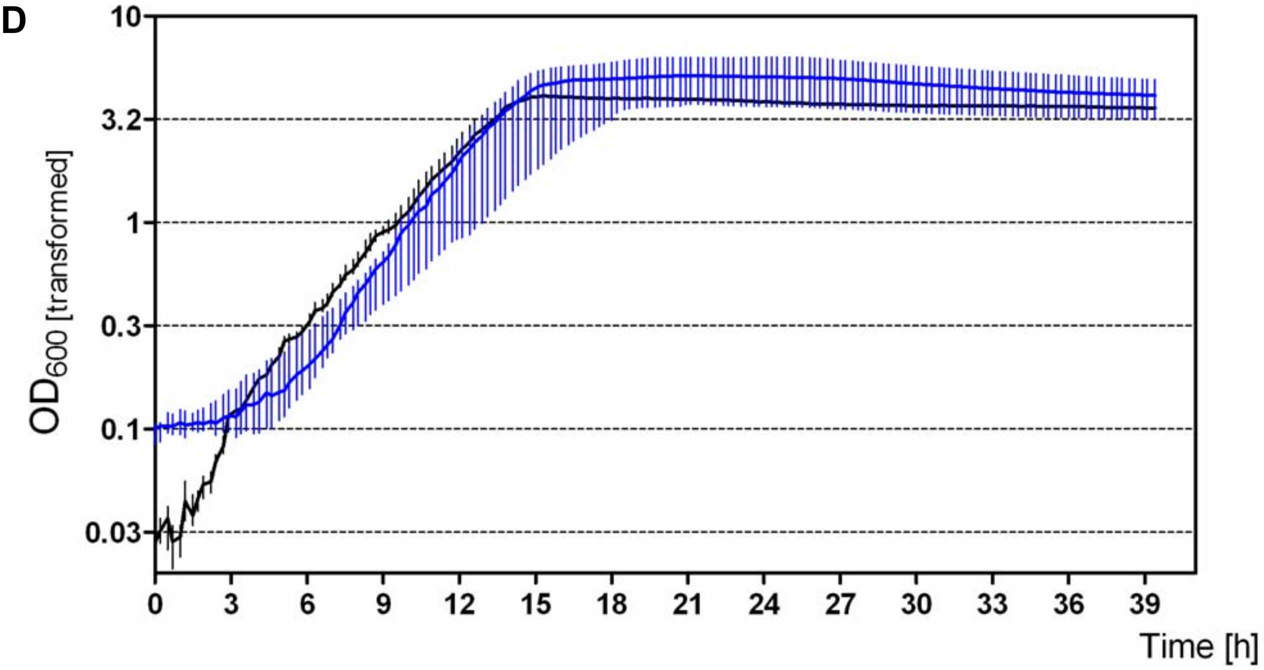

Figure S8. Restoration of wild type growth behaviour of *E. coli* Δ*kduID* containing complementing plasmids. *E. coli p*SU19 (black line) and *E. coli* Δ*kduID p*SU19-*kduID* (gray and blue line) were incubated in M9 minimal medium with 50mM glucuronate (A), 50mM glucuronate and 400 mM sucrose (B), 50mM galacturonate (C), or 50 mM galacturonate and 400 mM sucrose (D) under aerobic conditions. Cell densities were determined at 600 nm; data are expressed as medians and minima versus maxima (n = 6).
