# Supplementary material for: Novel Insights into E. coli’s Hexuronate Metabolism: KduI Facilitates the Conversion of Galacturonate and Glucuronate under Osmotic Stress Conditions
Source: PLoS One. 2013 Feb 21;8(2):e56906. doi: 10.1371/journal.pone.0056906 (PMC3578941; doi:10.1371/journal.pone.0056906)
Supplement: Table S1 — Specific activity of KduI and KduD, calculated for hexuronate concentrations observed after incubation of cell-free extracts of E. coli clones overexpressing kduI , kduD , or both genes with 10 mM galacturonate or glucuronate over 6 h at 37°C. (PDF) [file pone.0056906.s009.pdf]

Table S1. Specific activity of KduI and KduD, calculated for hexuronate concentrations observed after incubation of cell-free extracts of *E. coli* clones overexpressing *kduI*, *kduD*, or both genes with 10 mM galacturonate or glucuronate over 6 h at 37°C.

Medium	Specific activity after 2 h [nmol/min*mg] [a]				
	E. coli jm109 *p*GEM-T	*E. coli* jm109 *p*GEM-T-*kduID*	*E. coli* KRX *p*GEM-T	*E. coli* KRX *p*GEM-T-*kduD*	*E. coli* KRX *p*GEM-T-*kduI*
Glucuronate [10 mM]	1.8 (1.0:2.4)	5.2 (2.3:6.1) [b]	0.7 (-0.1:2.9)	1.2 (0.3:3.0)	3.7 (2.5:4.9) [b]
Galacturonate [10 mM]	1.6 (0.6:3.3)	3.1 (2.4:8.6) [b]	0.8 (-0.2:1.6)	1.3 (0.2:2.6)	4.4 (2.6:8.9) [b]

[a] Data are expressed as medians and minima versus maxima (n = 11-12).
[b] Data represent comparisons of the results obtained with *E. coli* JM109 *p*GEM-T vs. *E. coli* JM109 *p*GEM-T-*kduID*; *E. coli* KRX *p*GEM-T vs. *E. coli* KRX *p*GEM-T-*kduI* or *E. coli* KRX *p*GEM-T-*kduD* (Mann-Whitney test; P < 0.001).
