# Supplementary material for: Novel Insights into E. coli’s Hexuronate Metabolism: KduI Facilitates the Conversion of Galacturonate and Glucuronate under Osmotic Stress Conditions
Source: PLoS One. 2013 Feb 21;8(2):e56906. doi: 10.1371/journal.pone.0056906 (PMC3578941; doi:10.1371/journal.pone.0056906)
Supplement: Table S2 — Growth of E. coli MG1655 pSU19 and E. coli Δ kduID pSU19- kduID under aerobic conditions: Complementation of E. coli Δ kduID with plasmids containing the corresponding genes including physiologically relevant promoters restored wild type like behavior. (PDF) [file pone.0056906.s010.pdf]

Table S2: Growth of *E. coli* MG1655 pSU19 and *E. coli* Δ*kduID* pSU19-*kduID* under aerobic conditions: Complementation of *E. coli* Δ*kduID* with plasmids containing the corresponding genes including physiologically relevant promoters restored wild type like behavior.

	OD_{600} max.		Doubling time t_d (min^{-1})	
Medium [b]	*E. coli* MG1655 pSU19	*E. coli* Δ*kduID* pSU19-*kduID*	*E. coli* MG1655 pSU19	*E. coli* Δ*kduID* pSU19-*kduID*
Glucuronate	3.6 (3.4:3.8)	4.2 (4.0:4.4) [c]	89 (75:113)	82 (80:93)
Glucuronate, sucrose [400 mM]	3.4 (3.0:3.8)	4.2 (3.8:4.4) [c]	218 (183:226)	237 (195:271)
Galacturonate	5.4 (5.3:5.4)	5.3 (5.2:5.4)	78 (74:82)	82 (75:86)
galacturonate, sucrose [400 mM]	4.1 (4.0:4.3)	5.2 (3.7:6.3)	122 (115:125)	134 (94:208)

[a] Data are expressed as medians and minima versus maxima (n = 6).
[b] Cultures were incubated on M9 minimal medium containing glucuronate or galacturonate [50 mM each] with or without 400 mM sucrose.
[c] Data represent comparisons of the results obtained with *E. coli* MG1655 versus *E. coli* Δ*kduID* that included use of the same medium (Mann-Whitney test; $P < 0.05$).
